# Supplementary material for: Heat shock factor 1 (HSF1) cooperates with estrogen receptor α (ERα) in the regulation of estrogen action in breast cancer cells
Source: eLife. 2021 Nov 16;10:e69843. doi: 10.7554/eLife.69843 (PMC8709578; doi:10.7554/eLife.69843)
Supplement: Supplementary file 6. [file elife-69843-supp6.docx]

**Supplementary File 6. RT-qPCR primers for gene expression analyses.**

| **Gene symbol** | **RefSeq** | **forward primer sequence** | **reverse primer sequence** |
| --- | --- | --- | --- |
|  |  |  |  |
| ***AMZ1*** | NM_001284355.3 | acagccgatagacctgagcga | tggagctggagcctgtcaga |
| ***AREG*** | NM_001657.4 | agagaccgagttgccccaga | tccagcagcataatggcctgagc |
| ***C1orf226*** | NM_001085375.2 | aggatgaaaggtcagtcctgcaa | tgcccaggtgatggctctgt |
| ***GREB1 v.C*** | NM_148903.2 | ttggcttggtgggaccagctt | ttctgccggaccaagggcta |
| ***HCK*** | NM_001172129.2 | tagaggaatccggggagtggt | cttccggctgatgcccttga |
| ***HSPA1A*** | NM_005345.5 | agctggagcaggtgtgtaaccc | aaaaacagcaatcttggaaaggccc |
| ***HSPB1*** | NM_001540.5 | acgcggaaatacacgctgcc | ttggcggcagtctcatcgga |
| ***HSPB8*** | NM_014365.2 | ggatacgtggaggtgtctggcaa | taaggagggacctggggagctt |
| ***HSPH1*** | NM_001286503.1 | aagttgaccagcctccagaa | tggtccacacagcttgtctc |
| ***IGFBP4*** | NM_001552.3 | cagccctctgacaaggacga | ctggtgctccggtctcgaat |
| ***KCNF1*** | NM_002236.5 | ccgcatgatggagctgacca | tgcgcttgagggcataggtg |
| ***LINC01016*** | NR_038989.1 | cgccgctgtcaagtcccatt | aaatgcagcccacccactcct |
| ***LRIG1*** | NM_001377344.1 | agccacagatcatcacccagc | cgcgtggacgtggacaaagt |
| ***RET*** | NM_001355216.1 | accacgcaaagtgatgtatgg | atctcctcgctgcagttgtc |
| ***SDK2*** | NM_001144952.2 | tgctaatccgatggcagccg | gctcaggttccgcatggagt |
| ***SMPD3*** | NM_018667.4 | cggctacttcgagtacatcctgt | tcccacctgcaccttgagaa |
| ***SMTNL2*** | NM_001114974.2 | aagtgggagcaggaaacggc | tgcaggtccacgtgctggta |
| ***WWC1*** | NM_001161661.1 | tgtgccggctgaatcggagt | acttgaccgaggaaggctgtgg |
| ***ACTB*** | NM_001101.5 | agagcctcgcctttgccgat | ttgcacatgccggagccgtt |
| ***GAPDH*** | NM_002046.7 | ttccatggcaccgtcaaggc | tgcaaatgagccccagccttct |
| ***HNRNPK*** | NM_002140.4 | atgctgtcctcattccactgac | cgcgacggtcatcaaacatca |
| ***HPRT1*** | NM_000194.3 | gccctggcgtcgtgattagt | tgatggcctcccatctcctt |
