# Supplementary material for: Heat shock factor 1 (HSF1) cooperates with estrogen receptor α (ERα) in the regulation of estrogen action in breast cancer cells
Source: eLife. 2021 Nov 16;10:e69843. doi: 10.7554/eLife.69843 (PMC8709578; doi:10.7554/eLife.69843)
Supplement: Supplementary file 7. [file elife-69843-supp7.docx]

**Supplementary File 7. ChIP-qPCR primers for ESR1 binding analyses.**

| **Gene symbol** | **RefSeq** | **forward primer sequence** | **reverse primer sequence** |
| --- | --- | --- | --- |
|  |  |  |  |
| ***AMZ1*** | NC_000007.14 | tttcttgcccaaaggccaggca | ctaagcaacaagggccgccaga |
| ***GREB1 (1)*** | NC_000002.12 | tggaggtcagctcagtcagtgt | gcgtcaagcaactacactcccac |
| ***GREB1 (2)*** | NC_000002.12 | agggcagagctgataacgtcct | agaatgacccagttgccacact |
| ***GREB1 (3)*** | NC_000002.12 | acctgtgtggagagcactgtgac | ggcaaatgccaccgtttcgtgt |
| ***IGFBP4*** | NC_000017.11 | acctggtgcttaggggtggat | cagggcacgctgacttcaacag |
| ***SDK2*** | NC_000017.11 | tcctgcatggcaggtctagtgc | gcatatttccaagccaggccagc |
| ***SMPD3*** | NC_000016.10 | gctggctcaccctggtcaca | tccaggccactgtggggaga |
| ***SMTNL2*** | NC_000017.11 | cccgggggaactttgatctggaa | agggtaaccgcttggtgtcc |
| *negative control locus* | NC_000012.12 | atggttgccactggggatct | tgccaaagcctaggggaaga |
