# Supplementary material for: Heat shock factor 1 (HSF1) cooperates with estrogen receptor α (ERα) in the regulation of estrogen action in breast cancer cells
Source: eLife. 2021 Nov 16;10:e69843. doi: 10.7554/eLife.69843 (PMC8709578; doi:10.7554/eLife.69843)
Supplement: Supplementary file 8. [file elife-69843-supp8.docx]

**Supplementary File 8. PCR primers for chromosome conformation capture assay.**

| **Gene symbol** | **RefSeq** | **primer** | **sequence** |
| --- | --- | --- | --- |
|  |  |  |  |
| ***HSPB8*** | NC_000012.12 | F1 | tacatcctccccagctcctc |
|  |  | R3 | gcccttgtatggactctcagg |
|  |  | R4 | tgagccgtatctacccagga |
| ***WWC1*** | NC_000005.10 | F4 | ggtgctggacatacaatagctc |
|  |  | R1 | ccataactcgctgttggaagc |
|  |  | R3 | gccacccaggcaaaggaaat |
|  |  | R5 | ccctaggaaccaaatggttgtg |
