# Supplementary material for: Heat shock factor 1 (HSF1) cooperates with estrogen receptor α (ERα) in the regulation of estrogen action in breast cancer cells
Source: eLife. 2021 Nov 16;10:e69843. doi: 10.7554/eLife.69843 (PMC8709578; doi:10.7554/eLife.69843)

**Blots for Figure 1-figure supplement 1A with relevant bands labeled.**

HSF1 left


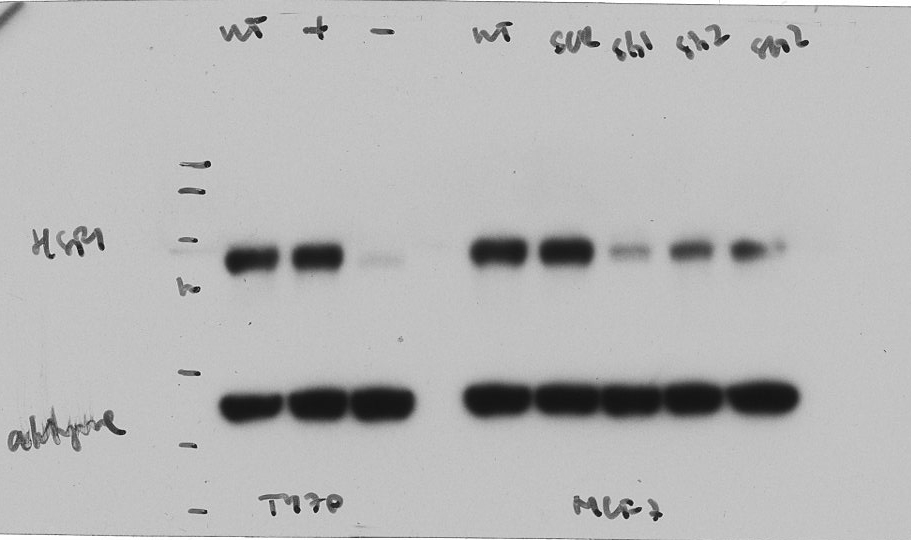


ACTB left


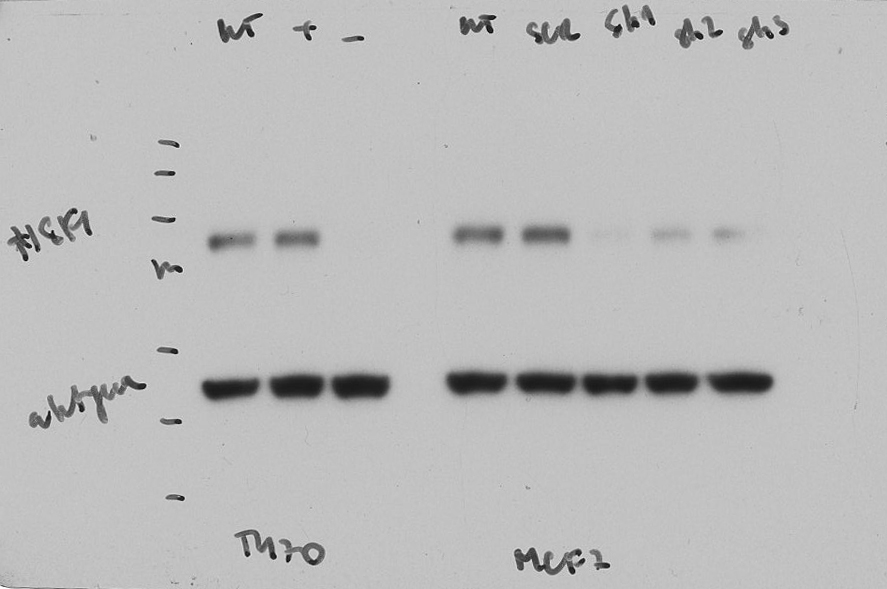


**
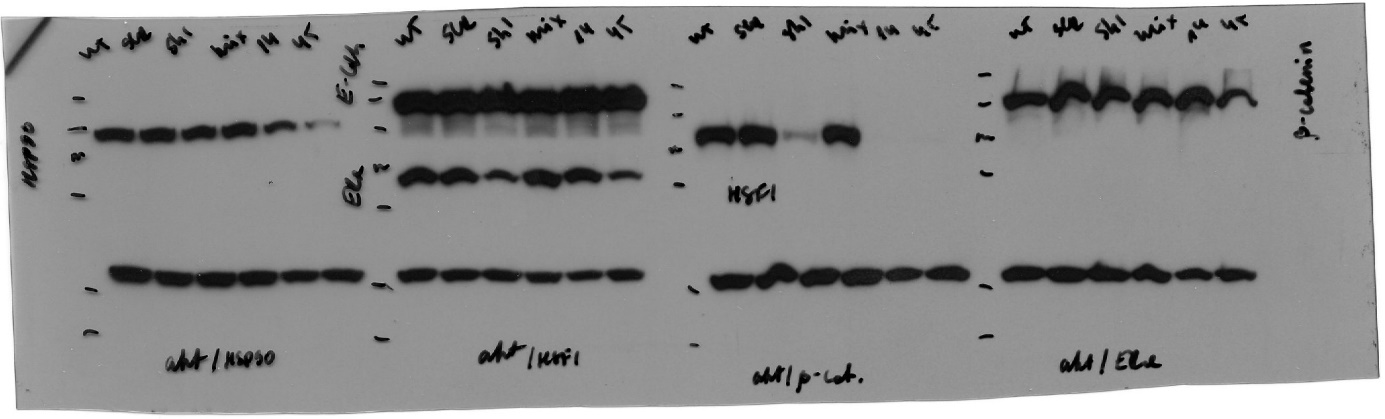
**HSF1 and ACTB right

**Blots for Figure 1-figure supplement 2A with relevant bands labeled.**

HSF1


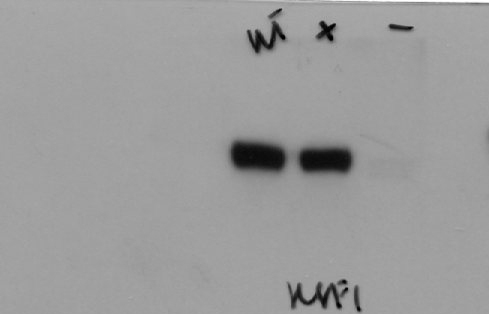


ACTB


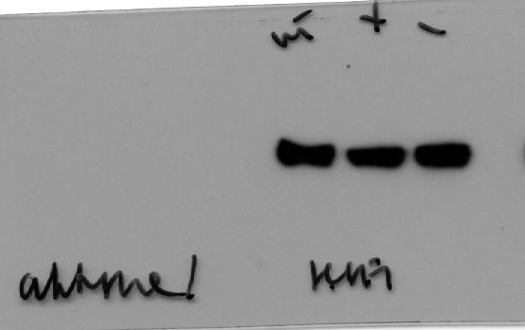


**Blots for Figure 3-figure supplement 2B with relevant bands labeled.**

**
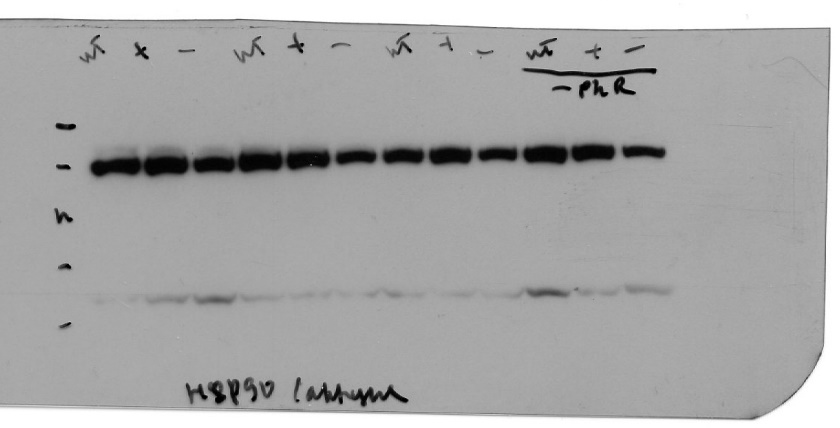
** MCF7 left (WT, HSF1+ and HSF1−), HSP90

**
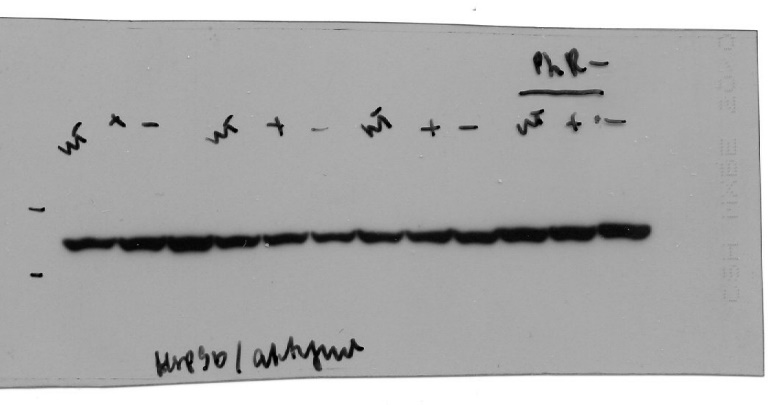
** MCF7 left, ACTB to HSP90

MCF7 right, HSP90 and ACTB


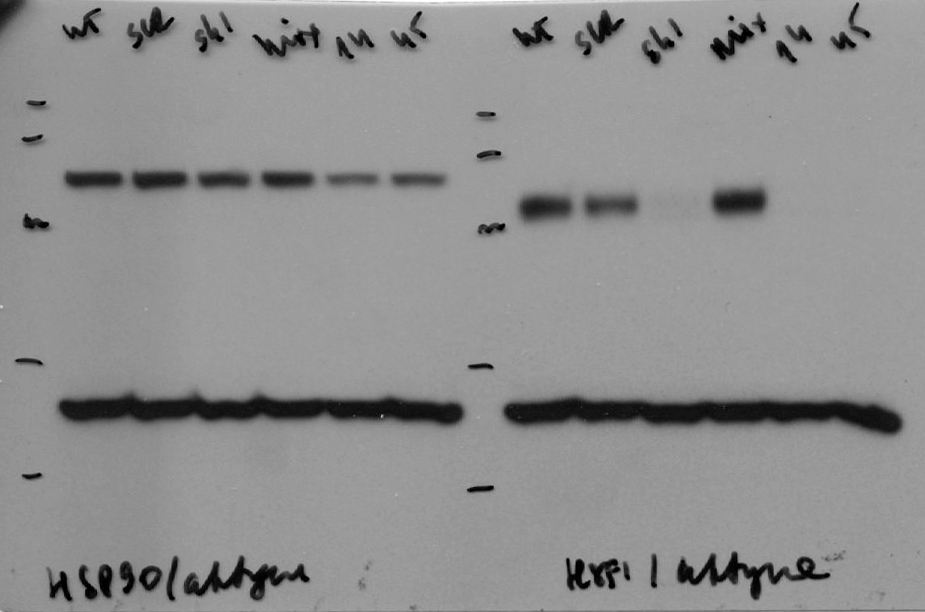


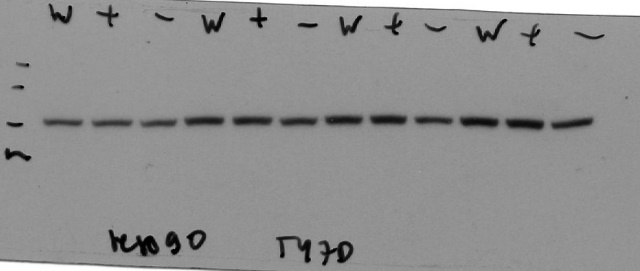
 T47D, HSP90


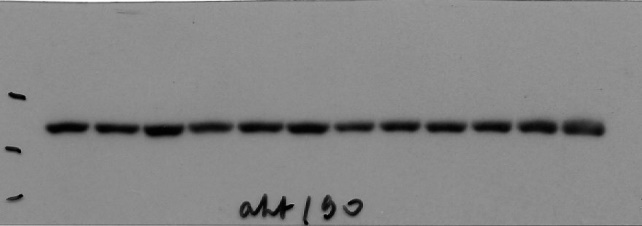
 T47D, ACTB to HSP90

**Blots for Figure 3-figure supplement 2C with relevant bands labeled.**

MCF7, ERα, and ACTB


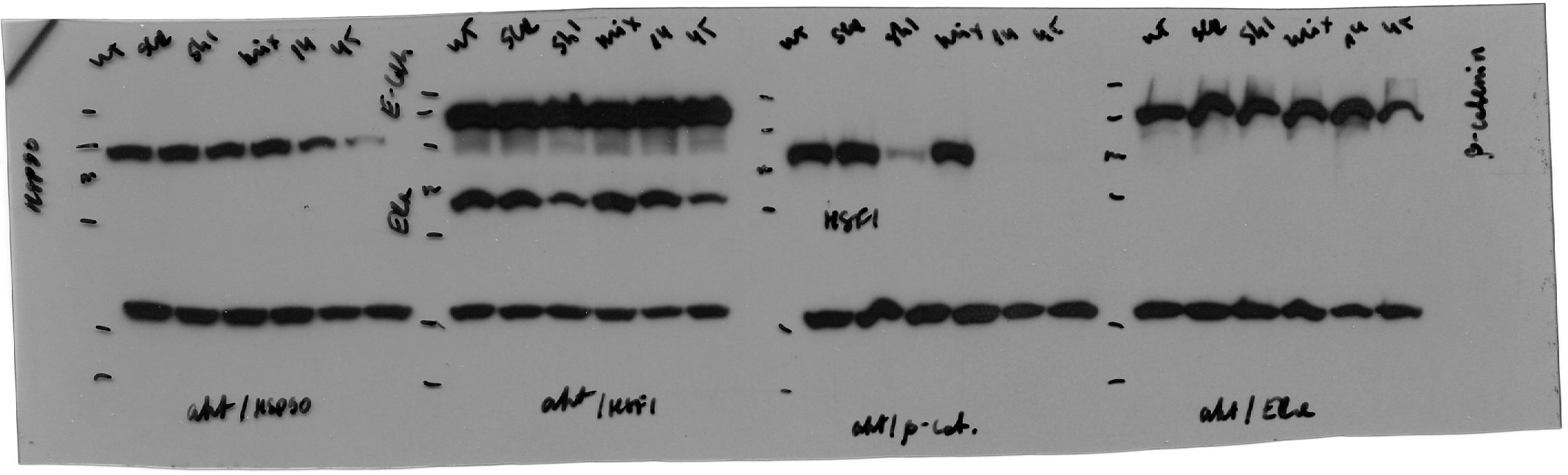


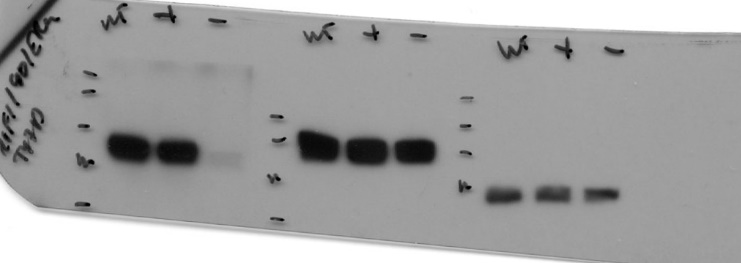
 T47D, ERα T47D, ACTB to ERα


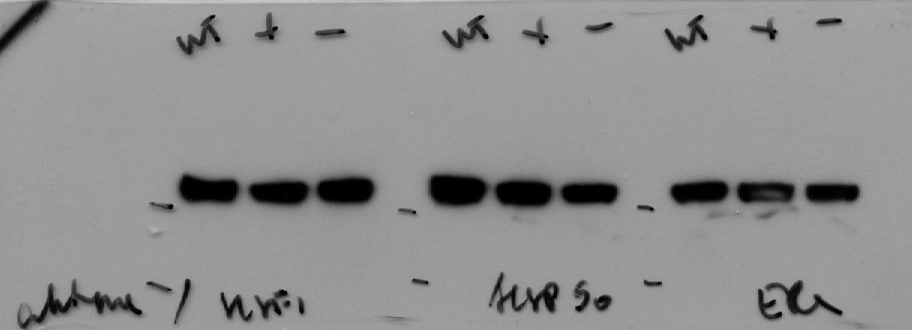

Supplement: Source data 1. — The original files of the full raw unedited blots and gels and figures with the uncropped blots and gels with the relevant bands labeled. [file elife-69843-supp9.zip › unprocessed blots_suppl_rev.docx]
