# Supplementary material for: Heat shock factor 1 (HSF1) cooperates with estrogen receptor α (ERα) in the regulation of estrogen action in breast cancer cells
Source: eLife. 2021 Nov 16;10:e69843. doi: 10.7554/eLife.69843 (PMC8709578; doi:10.7554/eLife.69843)

**Blots for Fig. 1A with relevant bands labeled.**

HSF1


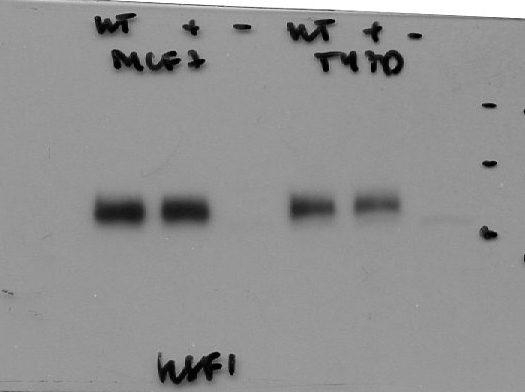


ACTB


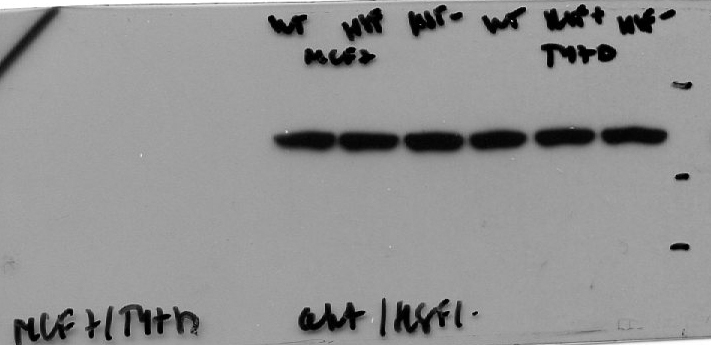


**Blots (G-box) for Fig. 1B with relevant bands labeled.**

HSP105


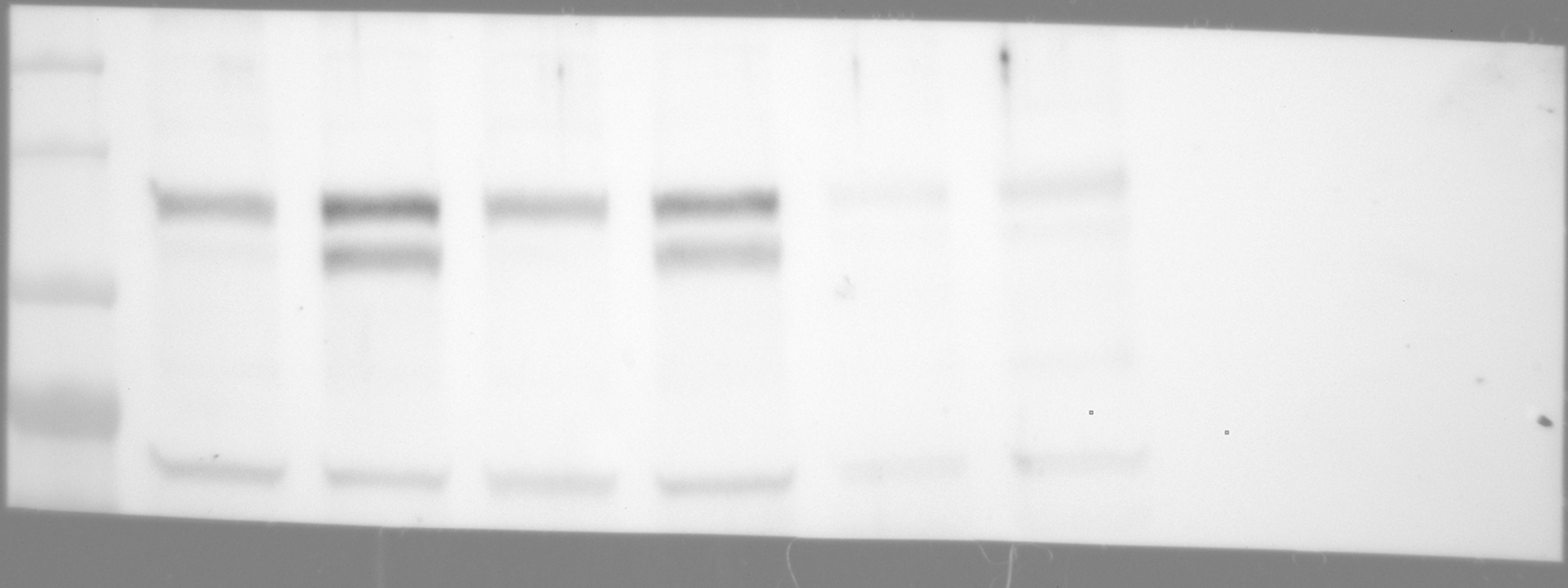


HSP90


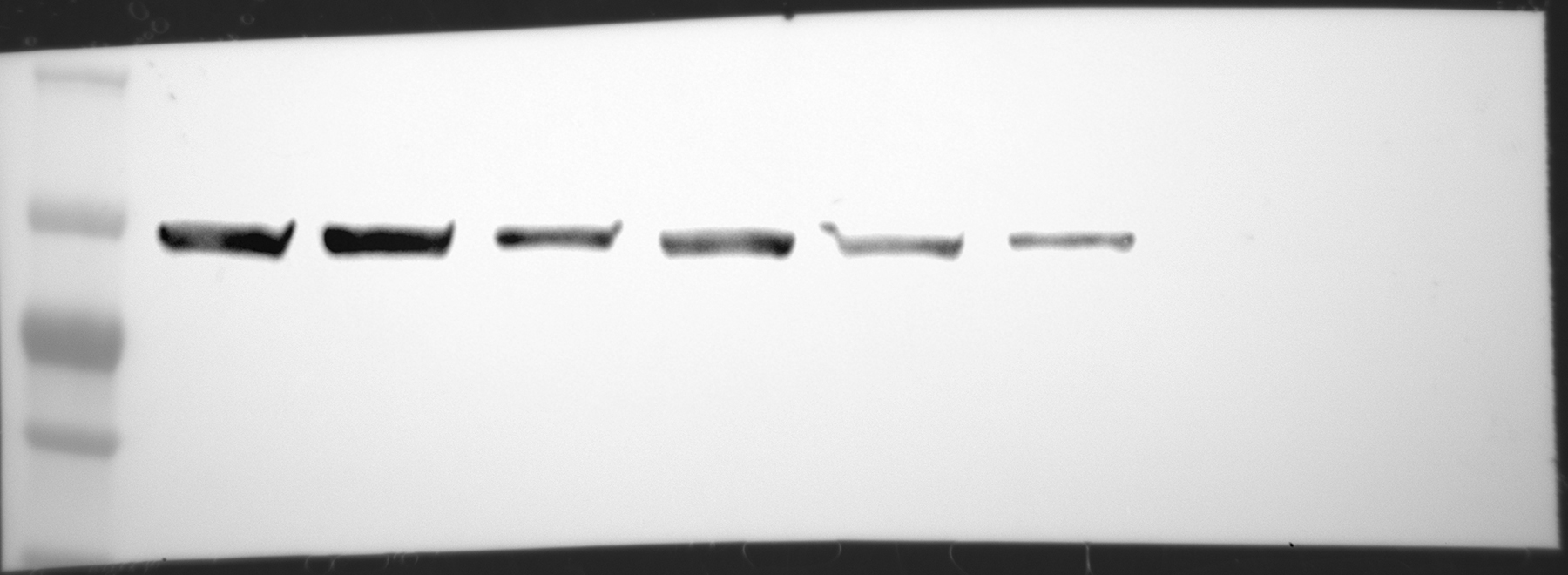


HSP70


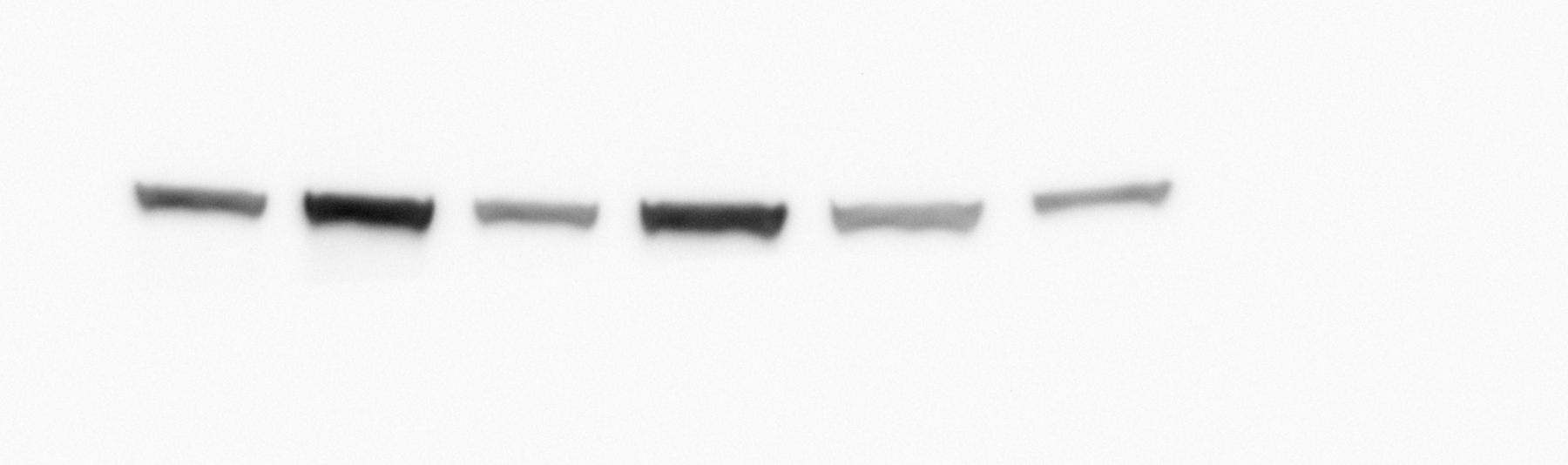

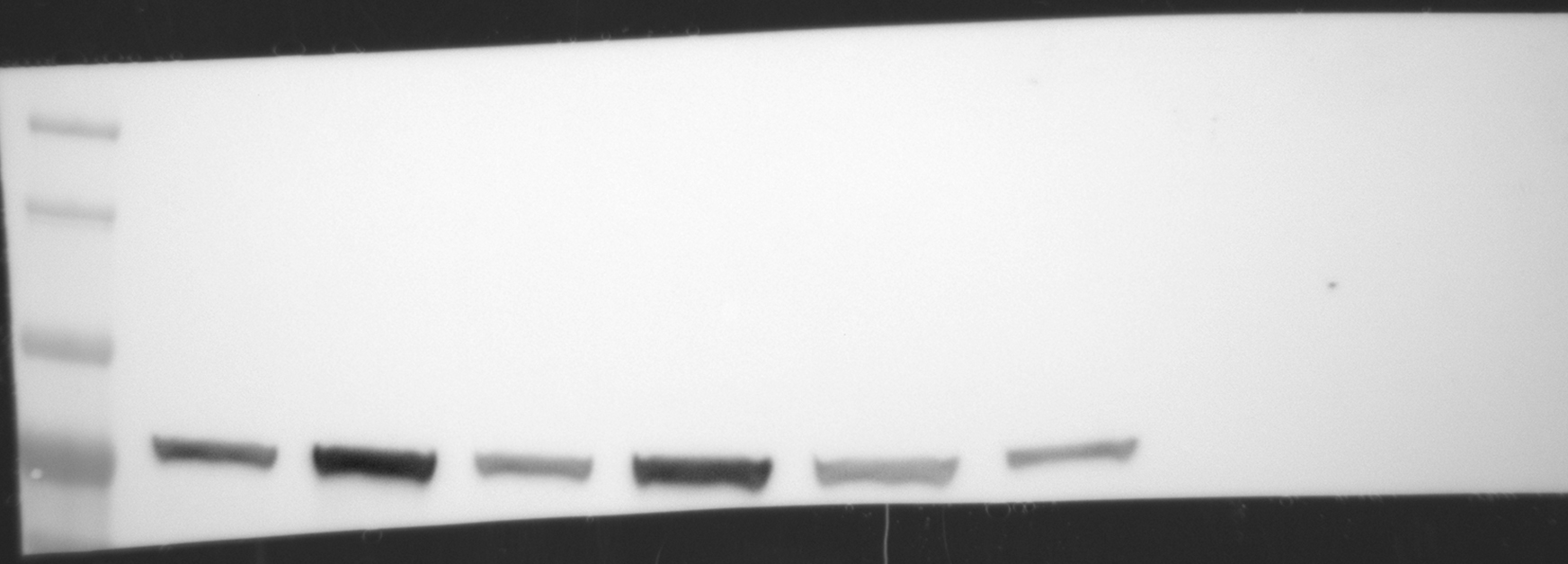


ACTB


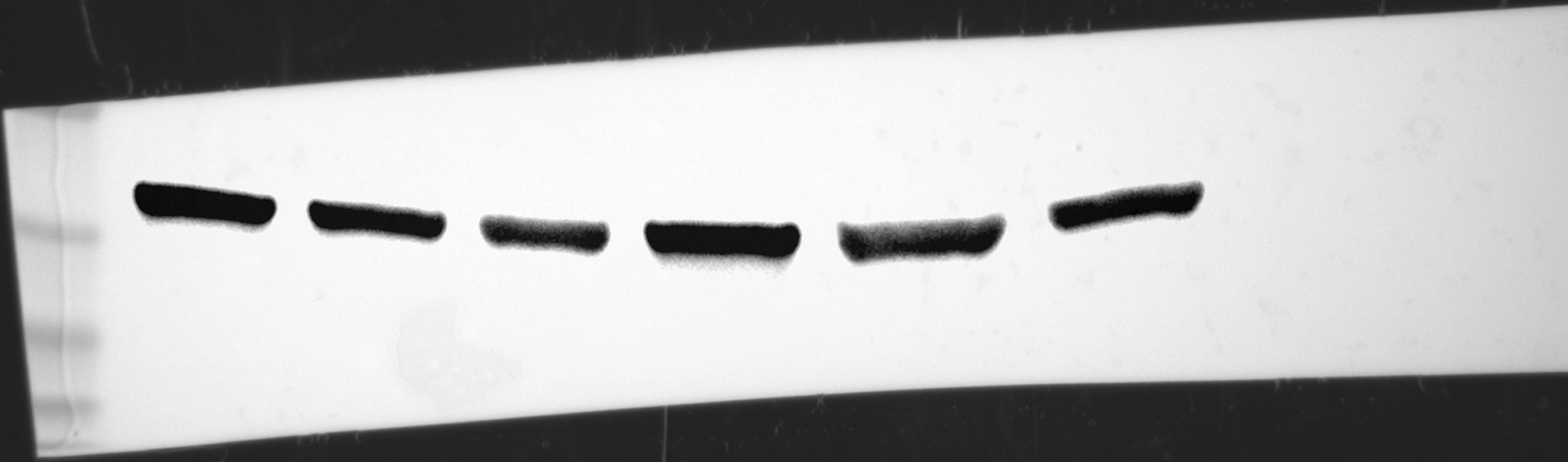


**Blots for Fig. 2E with relevant bands labeled.**


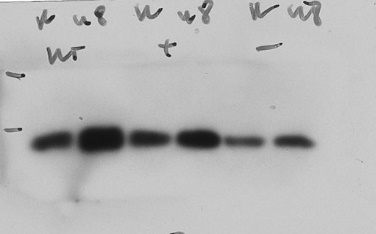
 HSPB8


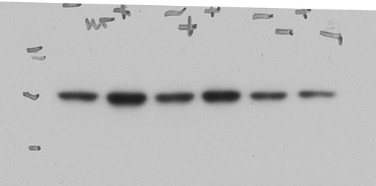
 PHLDA1


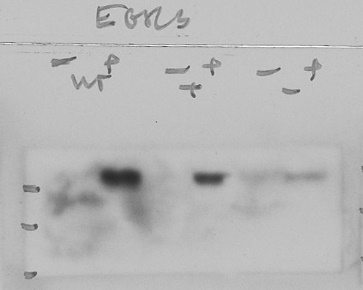
 EGR3


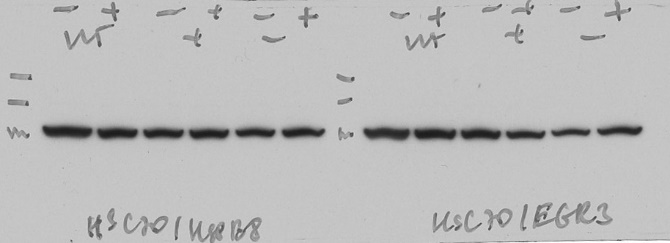
 HSPA8 toHSPB8 and EGR3


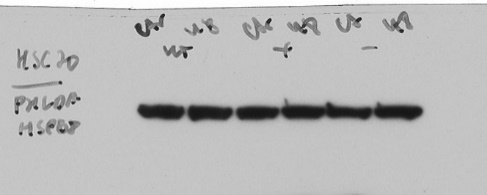
 HSPA8 to PHLDA1

**Blots for Fig. 3F with relevant bands labeled.**

ERα pS118


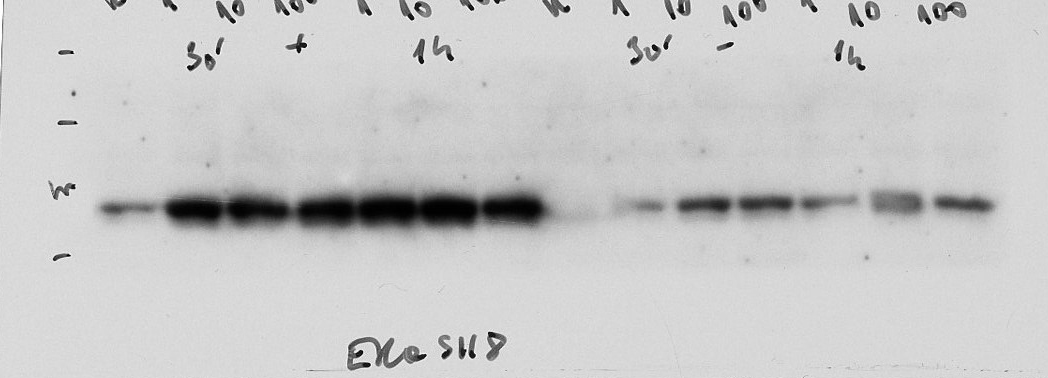


ACTB to ERα pS118


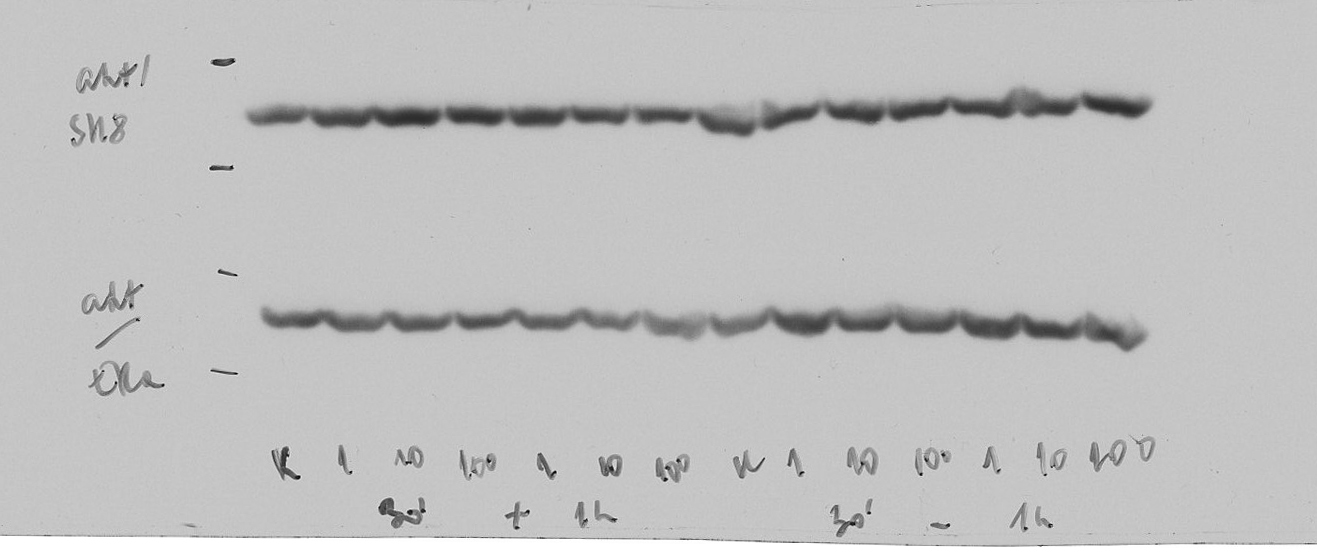


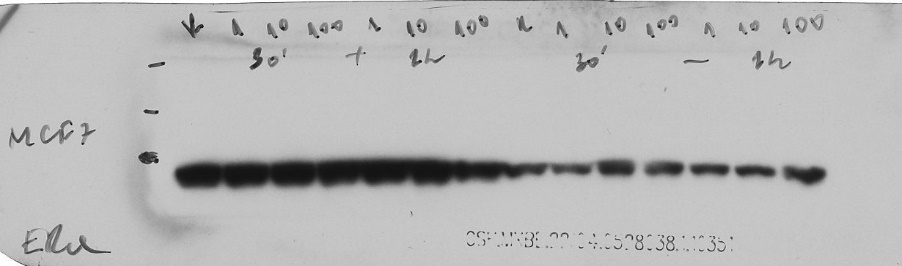
 ERα

ACTB to ERα


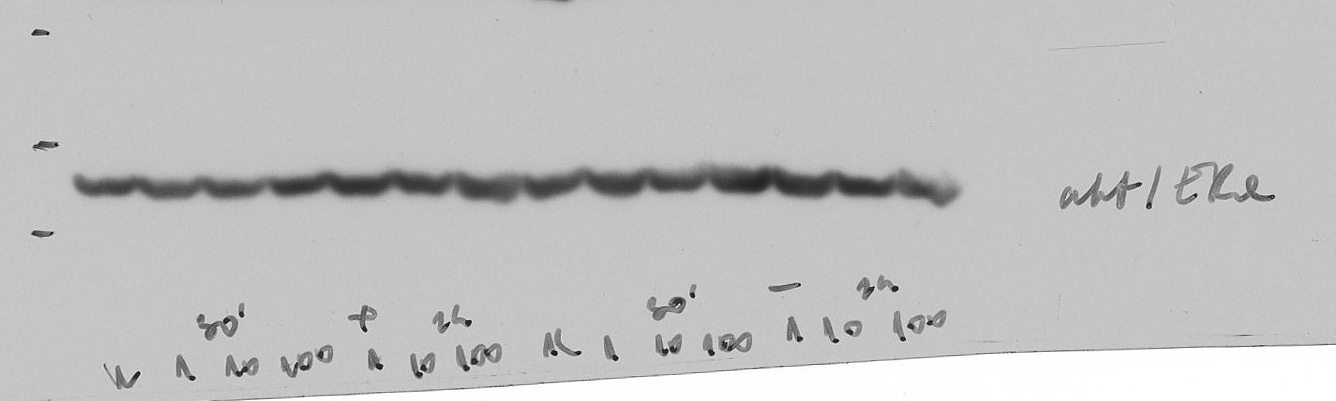


**Gels for Fig. 4E with relevant bands labeled.**

*HSPB8*, F1/R3


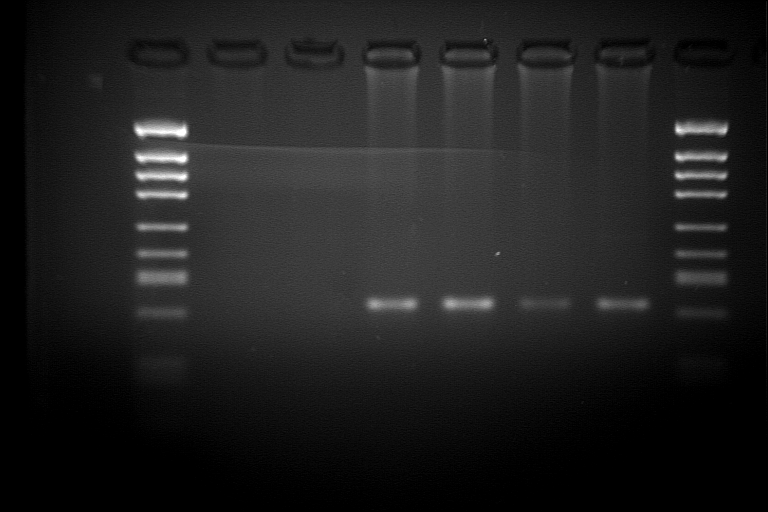


*HSPB8*, F1/R4


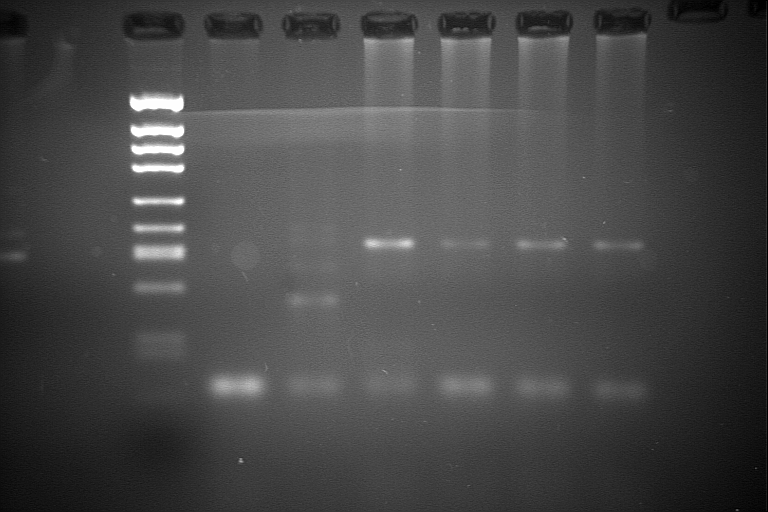


*WWC1*, R1/R3


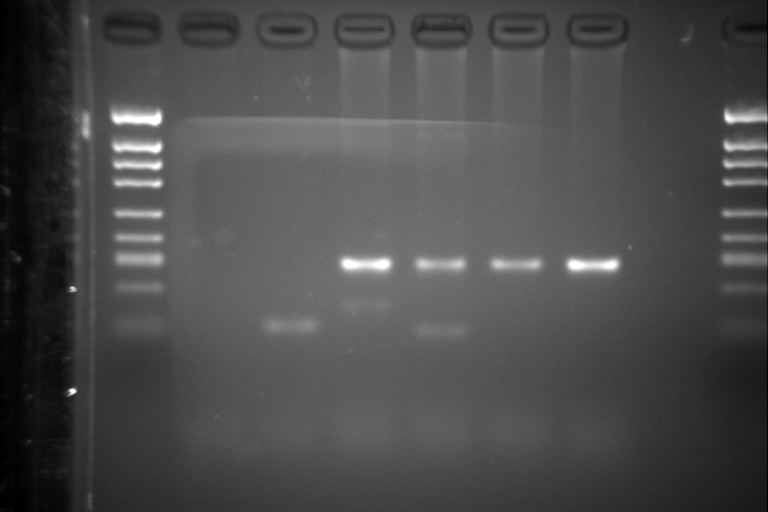


*WWC1*, R1/R5


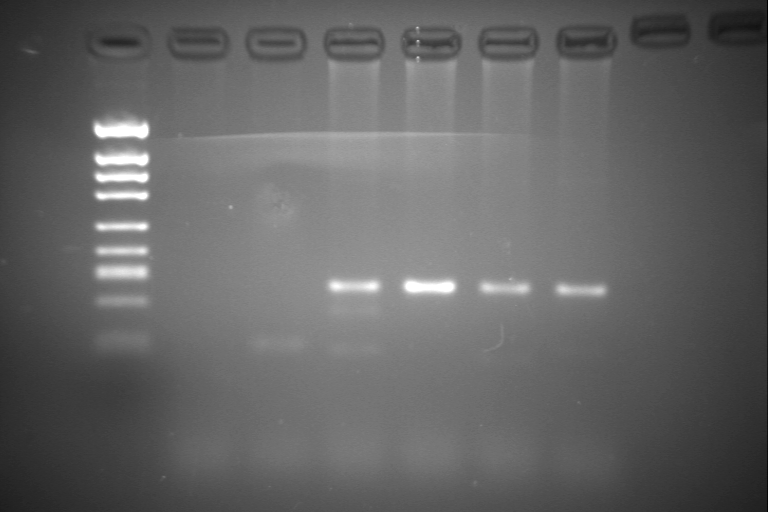


*WWC1*, F4/R3


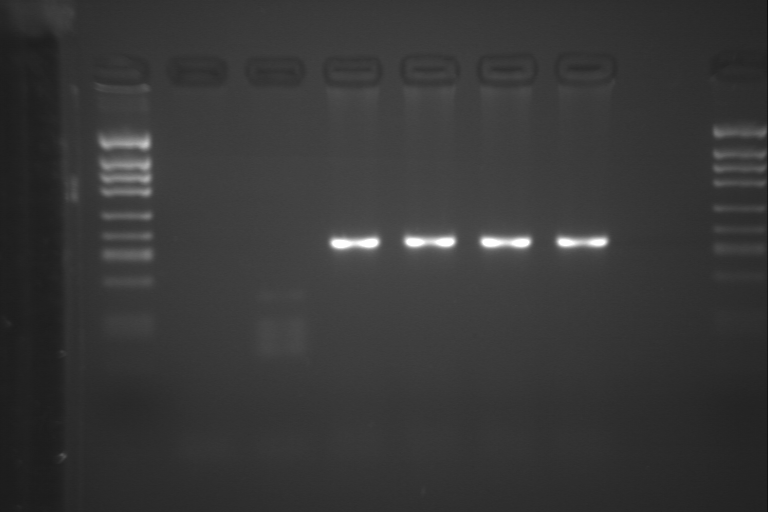


**Blots for Fig. 8B with relevant bands labeled.**

**
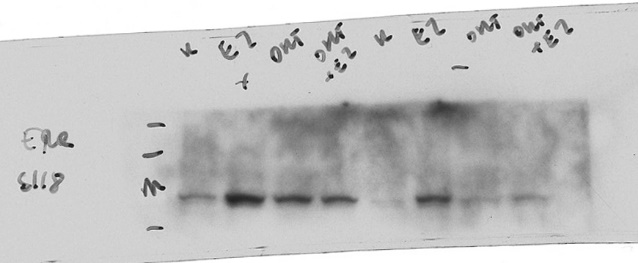
** ERα pS118

ACTB to ERα pS118


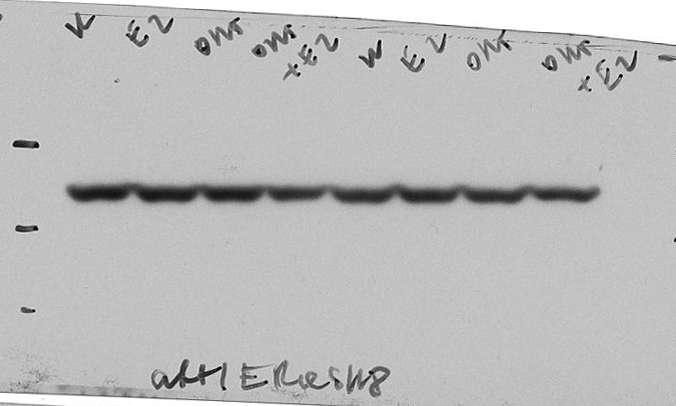


ERα


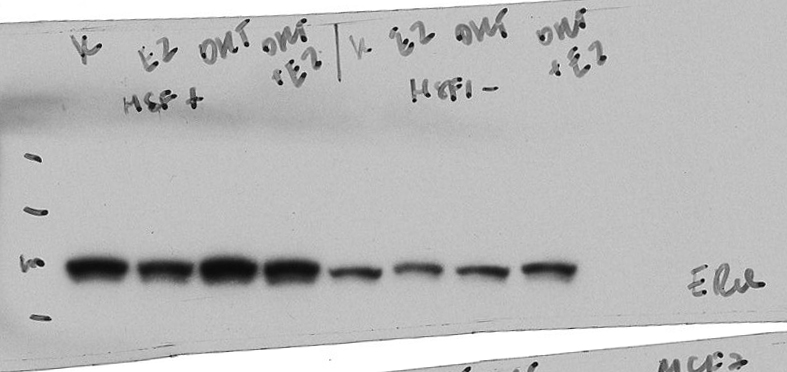


ACTB to ERα


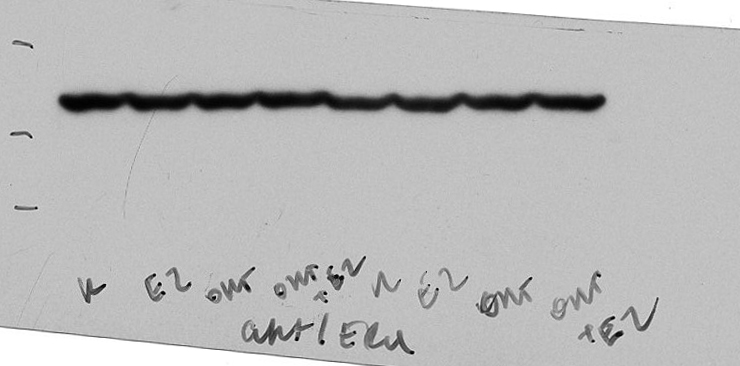

Supplement: Source data 1. — The original files of the full raw unedited blots and gels and figures with the uncropped blots and gels with the relevant bands labeled. [file elife-69843-supp9.zip › unprocessed gels and blots_rev.docx]
