# Supplementary figures and images for: Heat shock factor 1 (HSF1) cooperates with estrogen receptor α (ERα) in the regulation of estrogen action in breast cancer cells
Source: eLife. 2021 Nov 16;10:e69843. doi: 10.7554/eLife.69843 (PMC8709578; doi:10.7554/eLife.69843)

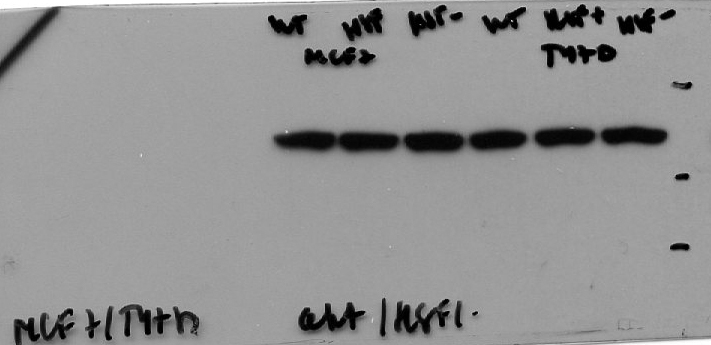

Supplement: Source data 1. — The original files of the full raw unedited blots and gels and figures with the uncropped blots and gels with the relevant bands labeled. [file elife-69843-supp9.zip › Fig 1A actin.jpg]

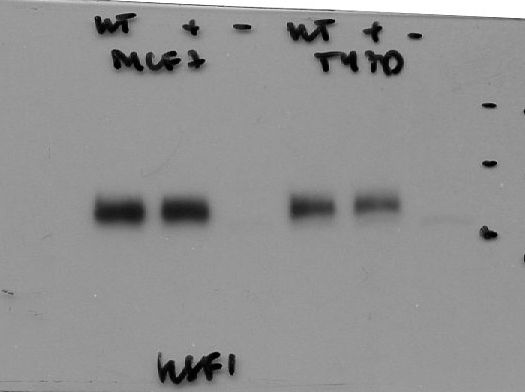

Supplement: Source data 1. — The original files of the full raw unedited blots and gels and figures with the uncropped blots and gels with the relevant bands labeled. [file elife-69843-supp9.zip › Fig 1A HSF1.jpg]

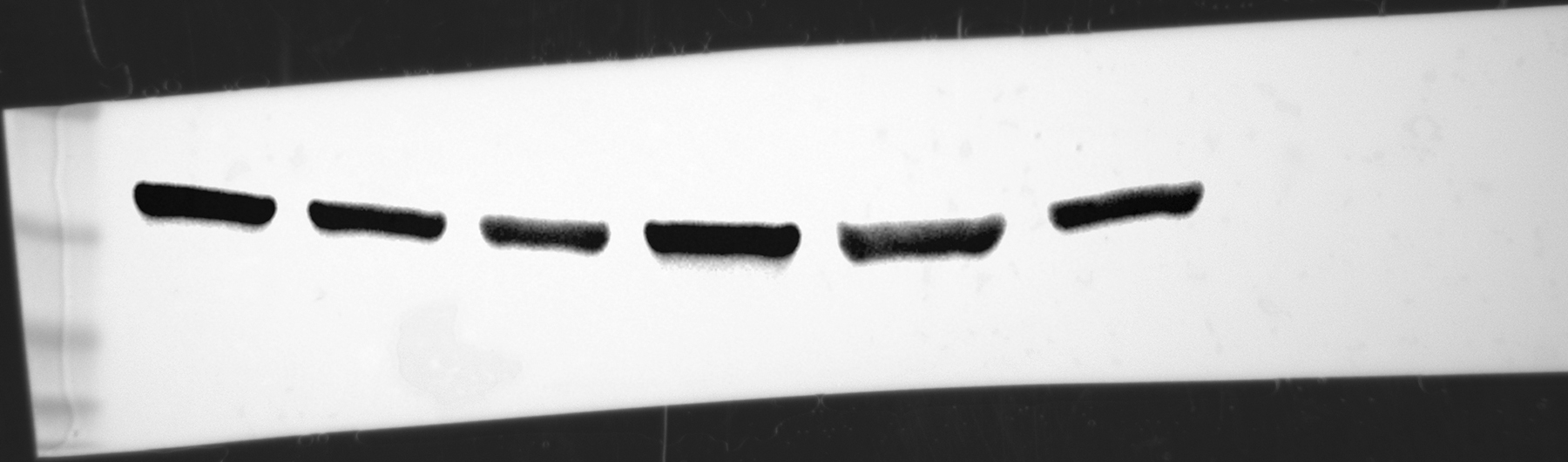

Supplement: Source data 1. — The original files of the full raw unedited blots and gels and figures with the uncropped blots and gels with the relevant bands labeled. [file elife-69843-supp9.zip › Fig 1B actin.jpg]

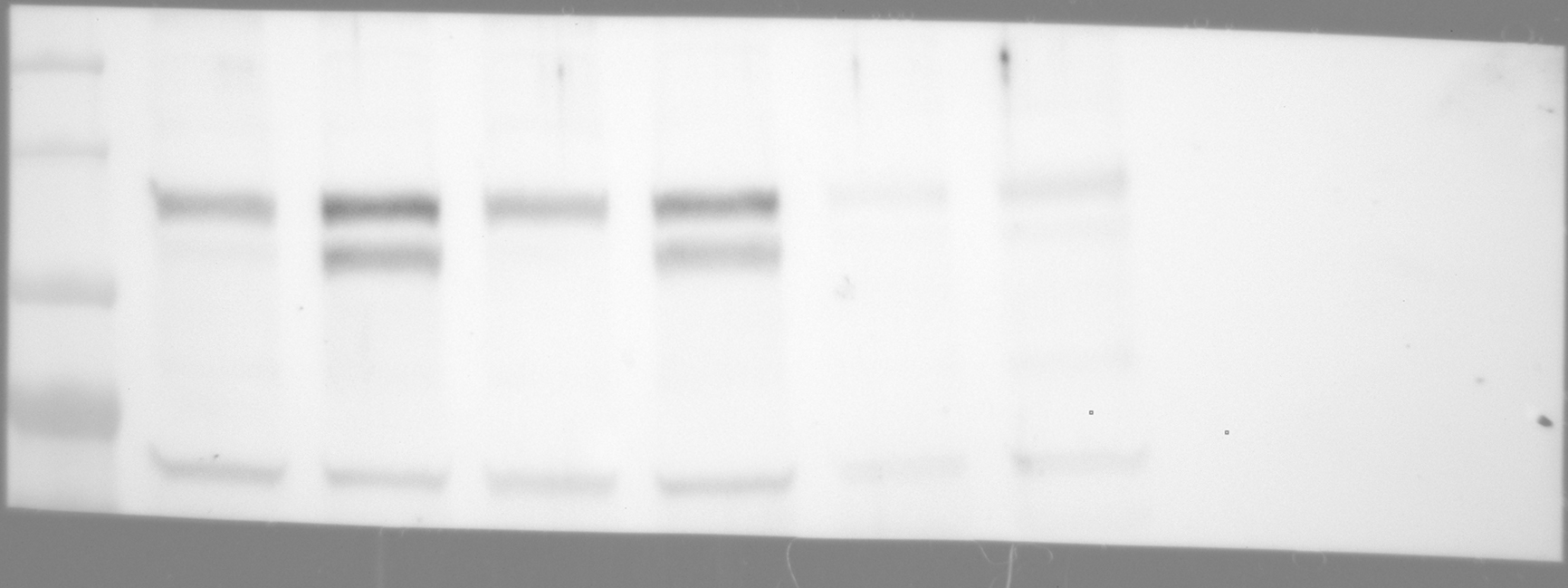

Supplement: Source data 1. — The original files of the full raw unedited blots and gels and figures with the uncropped blots and gels with the relevant bands labeled. [file elife-69843-supp9.zip › Fig 1B HSP105.jpg]

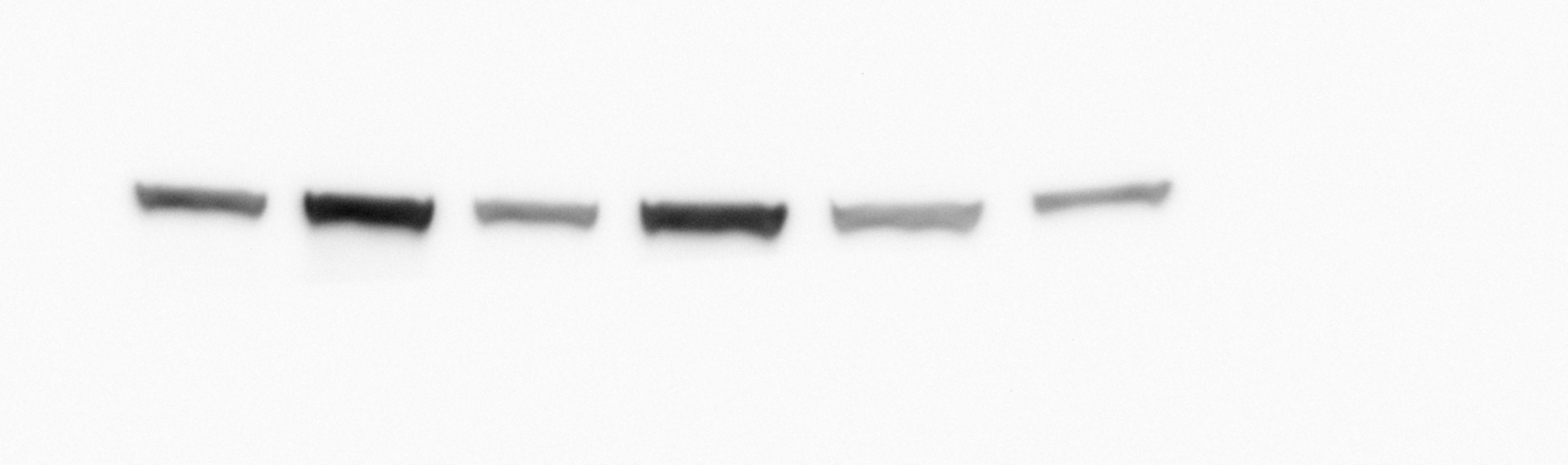

Supplement: Source data 1. — The original files of the full raw unedited blots and gels and figures with the uncropped blots and gels with the relevant bands labeled. [file elife-69843-supp9.zip › Fig 1B HSP70 invert.jpg]

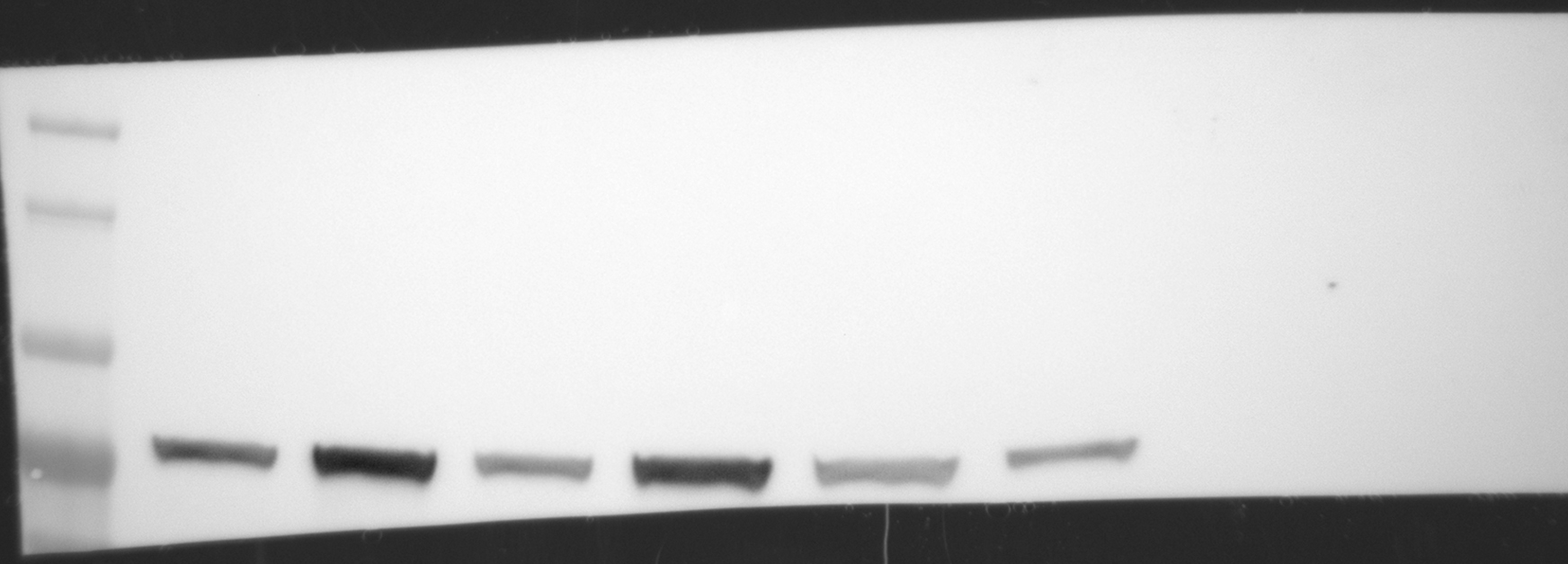

Supplement: Source data 1. — The original files of the full raw unedited blots and gels and figures with the uncropped blots and gels with the relevant bands labeled. [file elife-69843-supp9.zip › Fig 1B HSP70.jpg]

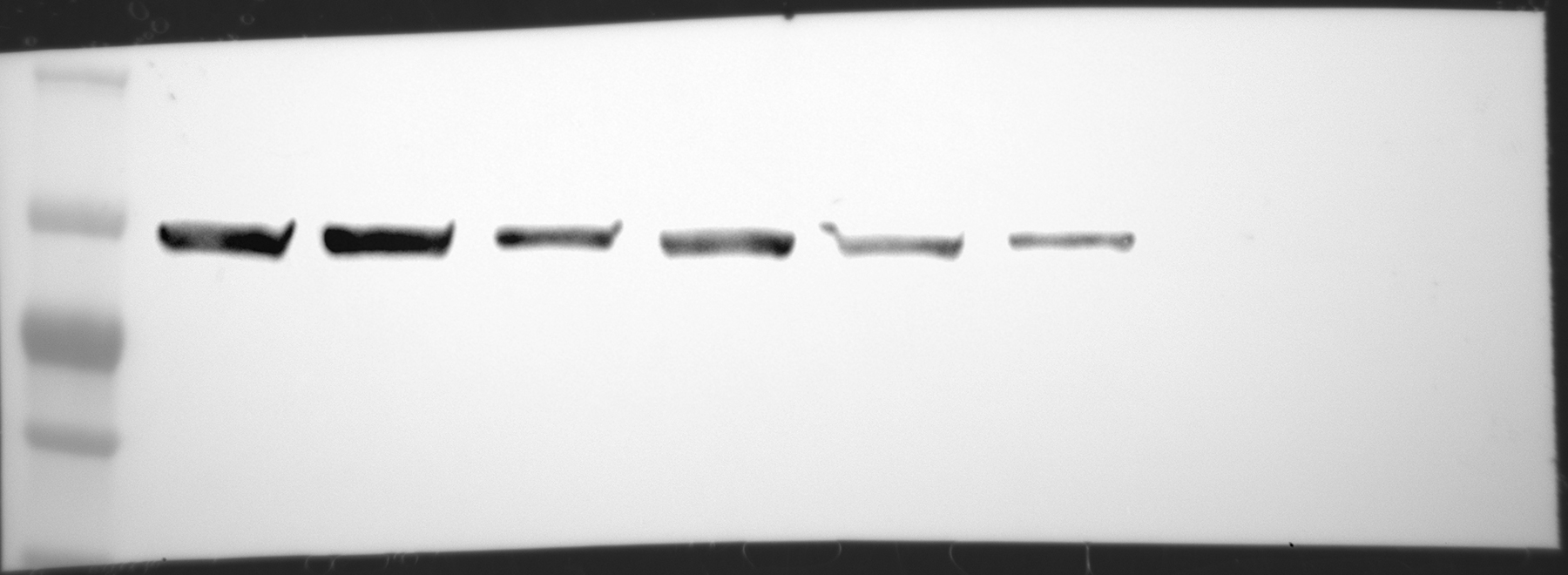

Supplement: Source data 1. — The original files of the full raw unedited blots and gels and figures with the uncropped blots and gels with the relevant bands labeled. [file elife-69843-supp9.zip › Fig 1B HSP90.jpg]

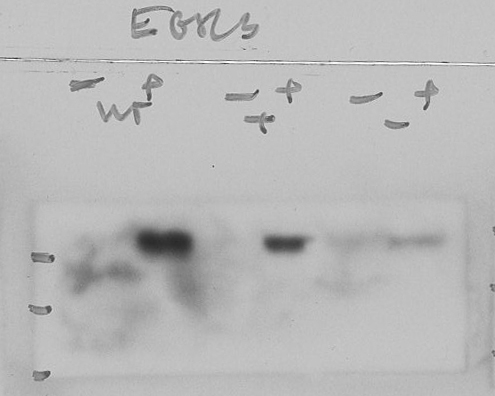

Supplement: Source data 1. — The original files of the full raw unedited blots and gels and figures with the uncropped blots and gels with the relevant bands labeled. [file elife-69843-supp9.zip › Fig 2E EGR3.jpg]

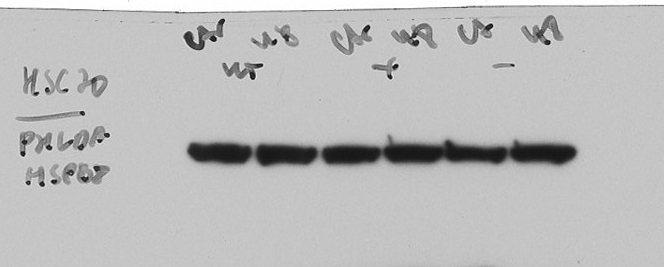

Supplement: Source data 1. — The original files of the full raw unedited blots and gels and figures with the uncropped blots and gels with the relevant bands labeled. [file elife-69843-supp9.zip › Fig 2E HSPA8 to HSPB8.jpg]

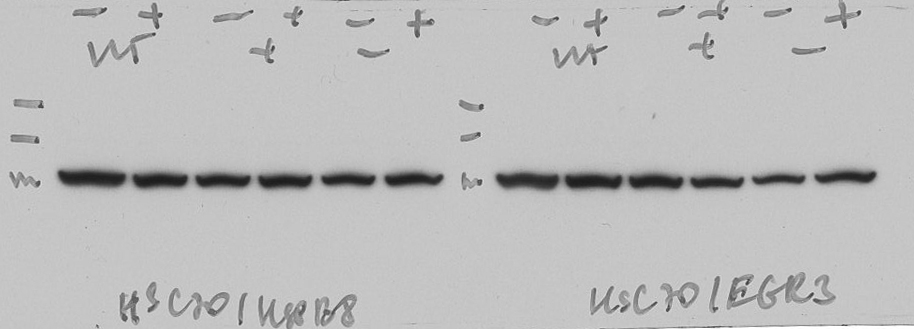

Supplement: Source data 1. — The original files of the full raw unedited blots and gels and figures with the uncropped blots and gels with the relevant bands labeled. [file elife-69843-supp9.zip › Fig 2E HSPA8 to PHLDA1 and EGR3.jpg]

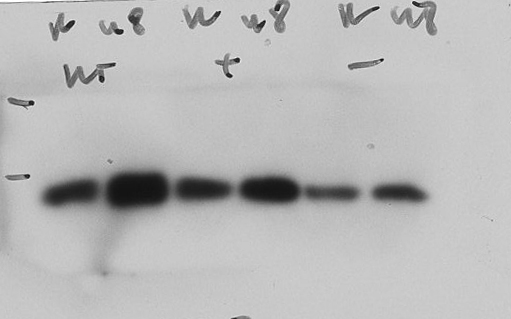

Supplement: Source data 1. — The original files of the full raw unedited blots and gels and figures with the uncropped blots and gels with the relevant bands labeled. [file elife-69843-supp9.zip › Fig 2E HSPB8.jpg]

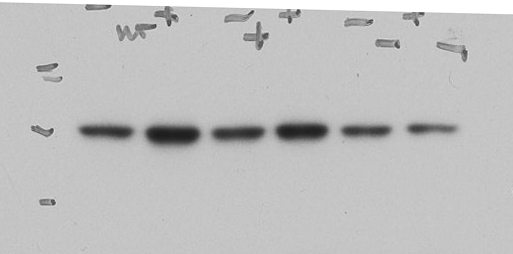

Supplement: Source data 1. — The original files of the full raw unedited blots and gels and figures with the uncropped blots and gels with the relevant bands labeled. [file elife-69843-supp9.zip › Fig 2E PHLDA1.jpg]

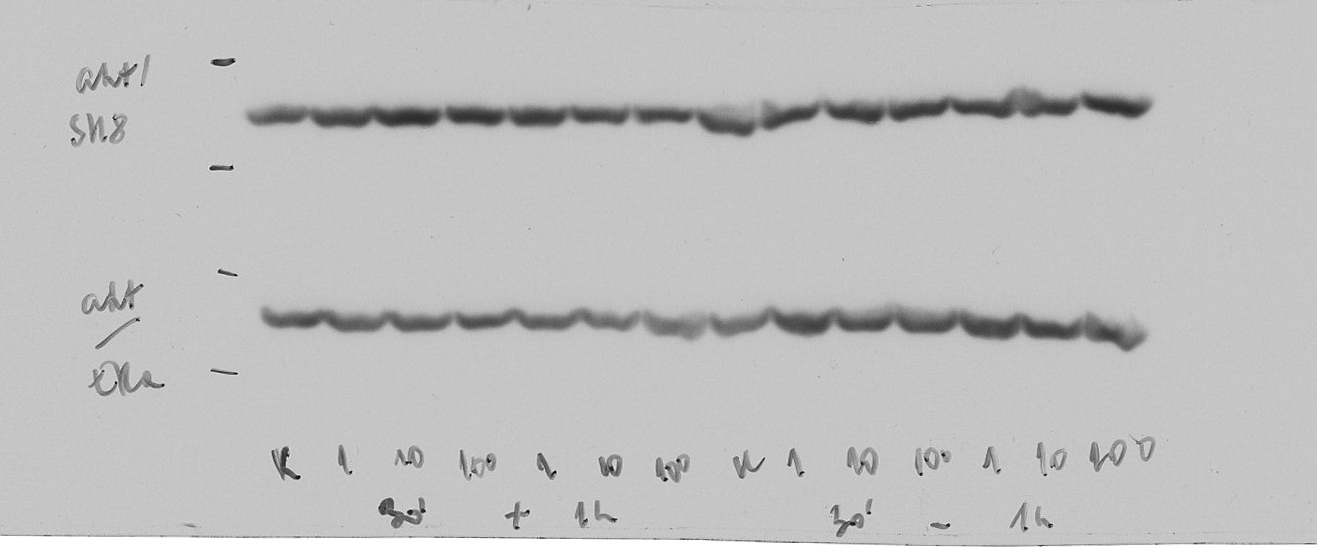

Supplement: Source data 1. — The original files of the full raw unedited blots and gels and figures with the uncropped blots and gels with the relevant bands labeled. [file elife-69843-supp9.zip › Fig 3F actin to ERalpha pS118.jpg]

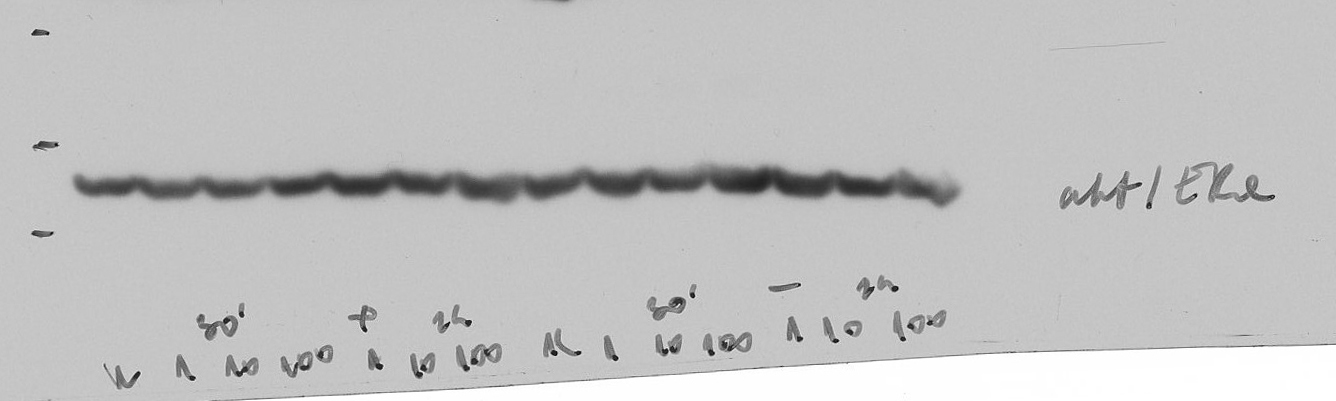

Supplement: Source data 1. — The original files of the full raw unedited blots and gels and figures with the uncropped blots and gels with the relevant bands labeled. [file elife-69843-supp9.zip › Fig 3F actin to ERalpha.jpg]

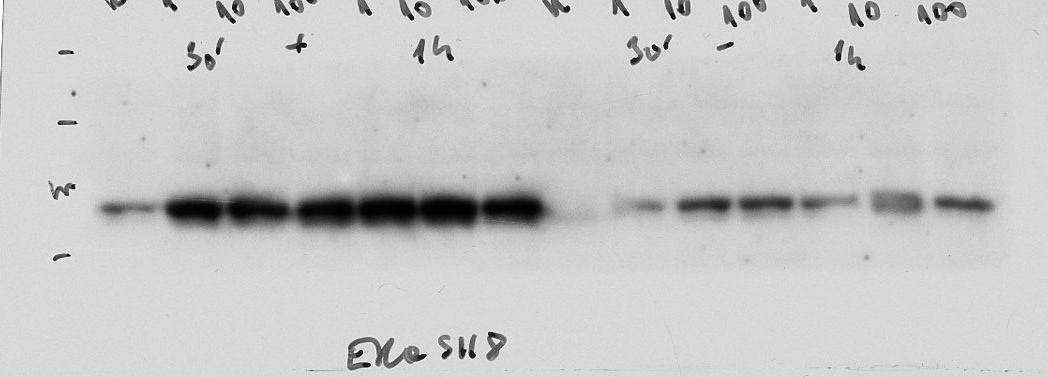

Supplement: Source data 1. — The original files of the full raw unedited blots and gels and figures with the uncropped blots and gels with the relevant bands labeled. [file elife-69843-supp9.zip › Fig 3F ERalpha pS118.jpg]

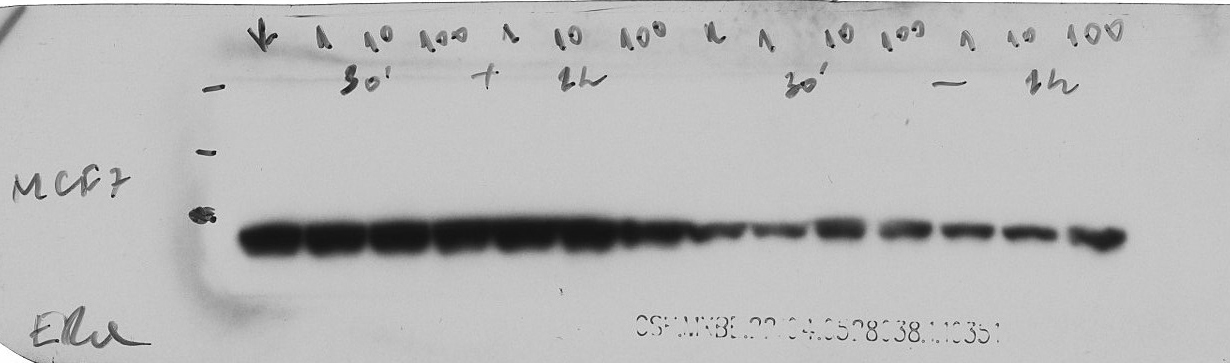

Supplement: Source data 1. — The original files of the full raw unedited blots and gels and figures with the uncropped blots and gels with the relevant bands labeled. [file elife-69843-supp9.zip › Fig 3F ERalpha.jpg]

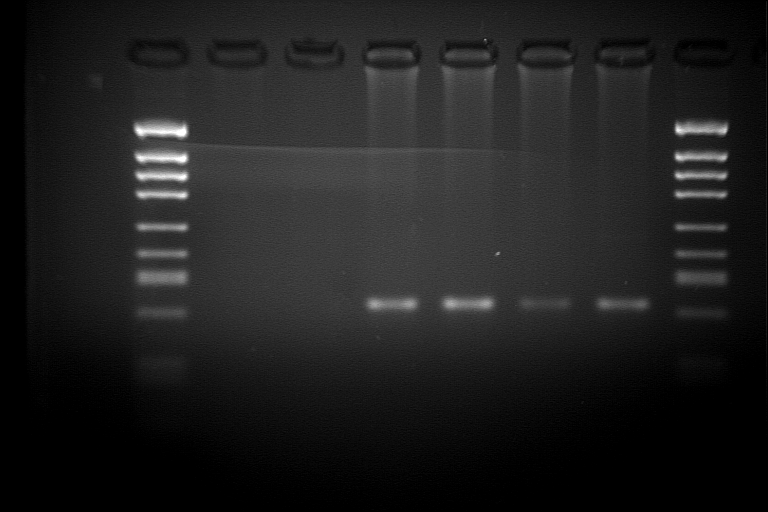

Supplement: Source data 1. — The original files of the full raw unedited blots and gels and figures with the uncropped blots and gels with the relevant bands labeled. [file elife-69843-supp9.zip › Fig 4E_HSPB8 F1R3.bmp]

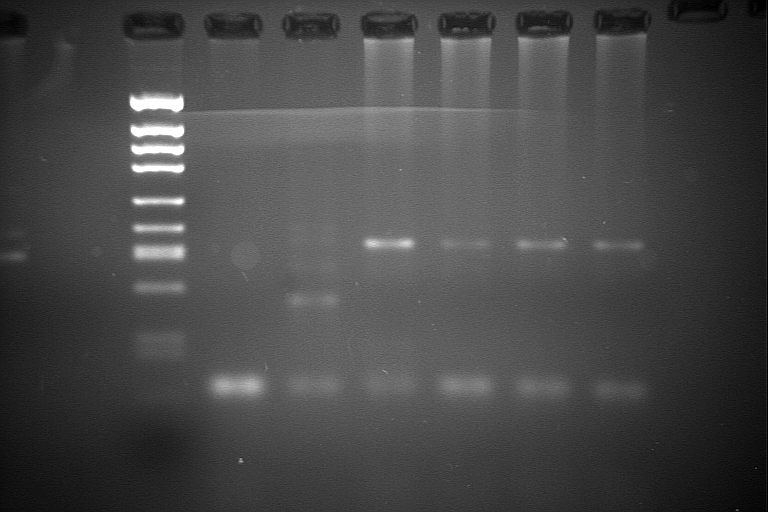

Supplement: Source data 1. — The original files of the full raw unedited blots and gels and figures with the uncropped blots and gels with the relevant bands labeled. [file elife-69843-supp9.zip › Fig 4E_HSPB8 F1R4.bmp]

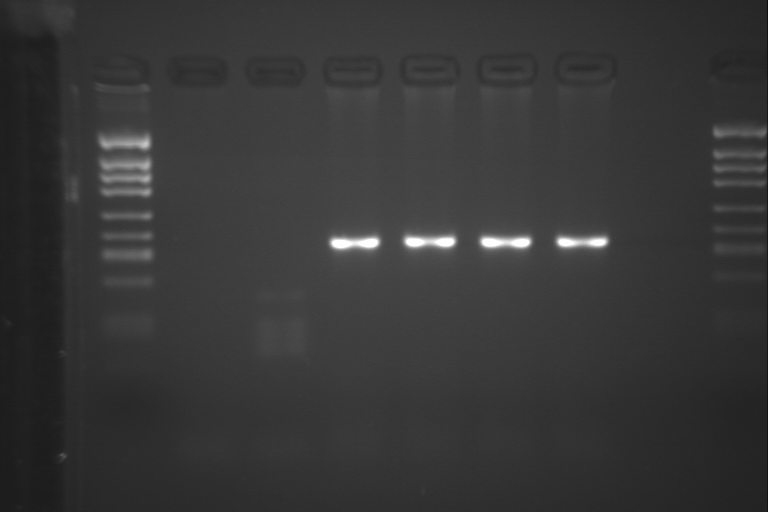

Supplement: Source data 1. — The original files of the full raw unedited blots and gels and figures with the uncropped blots and gels with the relevant bands labeled. [file elife-69843-supp9.zip › Fig 4E_WWC1 F4R3.bmp]

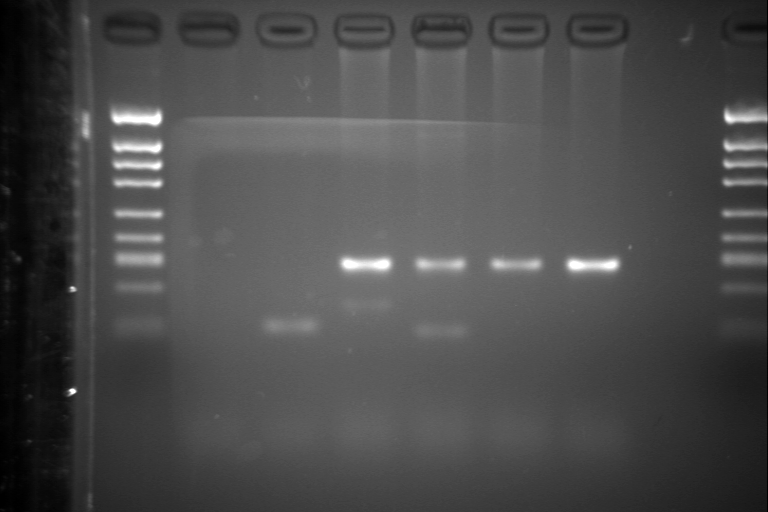

Supplement: Source data 1. — The original files of the full raw unedited blots and gels and figures with the uncropped blots and gels with the relevant bands labeled. [file elife-69843-supp9.zip › Fig 4E_WWC1 R1R3.bmp]

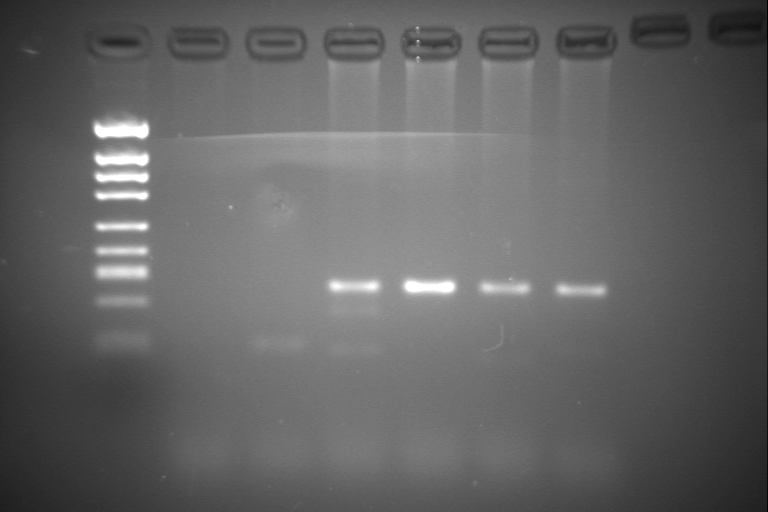

Supplement: Source data 1. — The original files of the full raw unedited blots and gels and figures with the uncropped blots and gels with the relevant bands labeled. [file elife-69843-supp9.zip › Fig 4E_WWC1 R1R5.bmp]

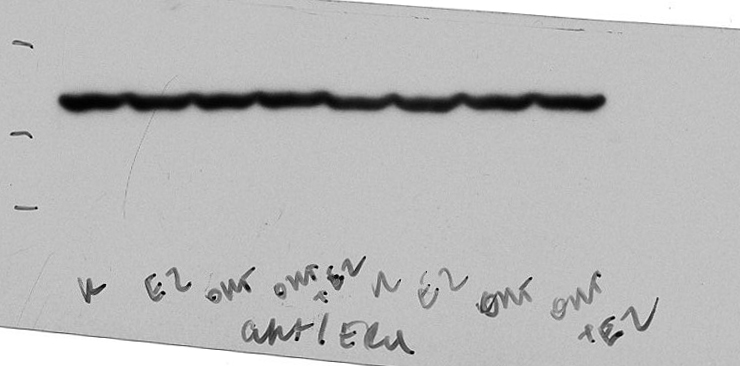

Supplement: Source data 1. — The original files of the full raw unedited blots and gels and figures with the uncropped blots and gels with the relevant bands labeled. [file elife-69843-supp9.zip › Fig 8B act ERalpha 4'OHT.jpg]

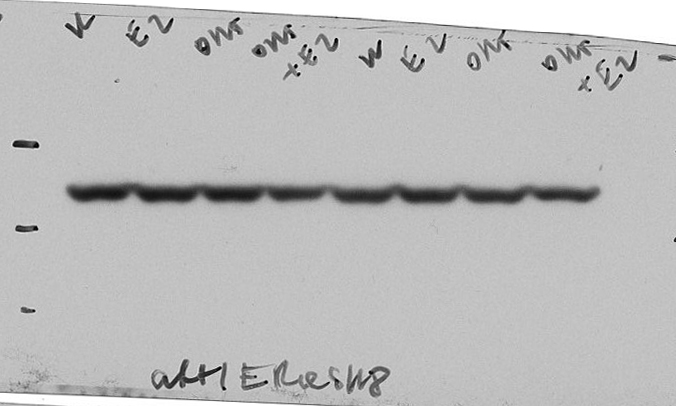

Supplement: Source data 1. — The original files of the full raw unedited blots and gels and figures with the uncropped blots and gels with the relevant bands labeled. [file elife-69843-supp9.zip › Fig 8B act ERalpha pS118 4'OHT.jpg]

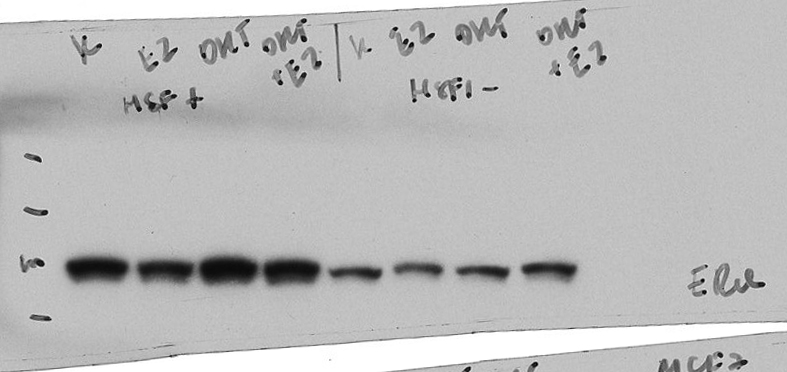

Supplement: Source data 1. — The original files of the full raw unedited blots and gels and figures with the uncropped blots and gels with the relevant bands labeled. [file elife-69843-supp9.zip › Fig 8B ERalpha 4'OHT.jpg]

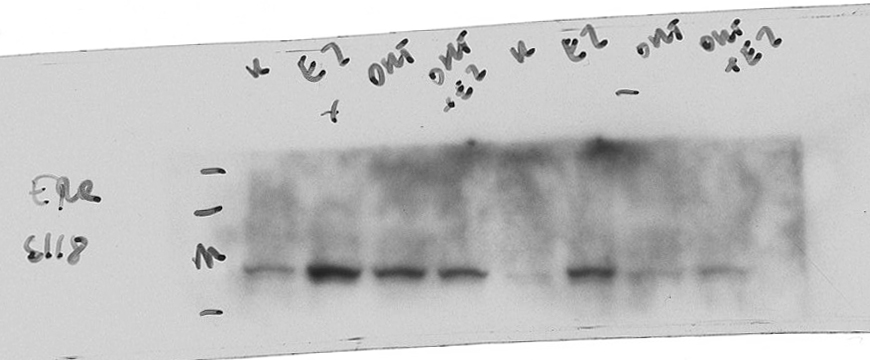

Supplement: Source data 1. — The original files of the full raw unedited blots and gels and figures with the uncropped blots and gels with the relevant bands labeled. [file elife-69843-supp9.zip › Fig 8B ERalpha pS118 4'OHT.jpg]

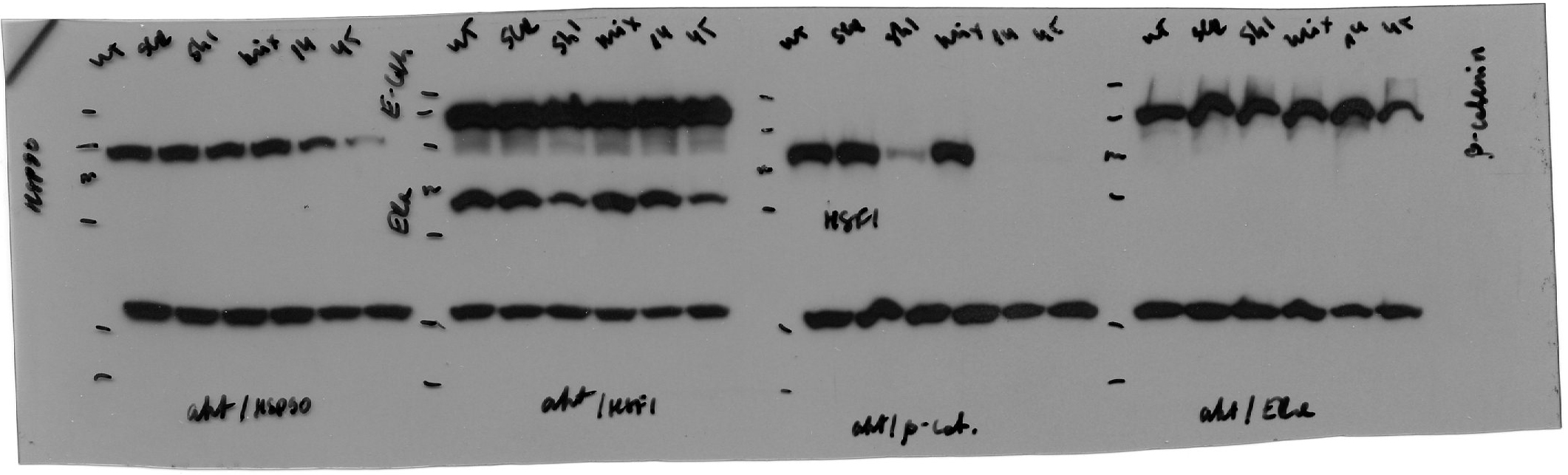

Supplement: Source data 1. — The original files of the full raw unedited blots and gels and figures with the uncropped blots and gels with the relevant bands labeled. [file elife-69843-supp9.zip › Figure 1-figure supplement 1A HSF1 actin.jpg]

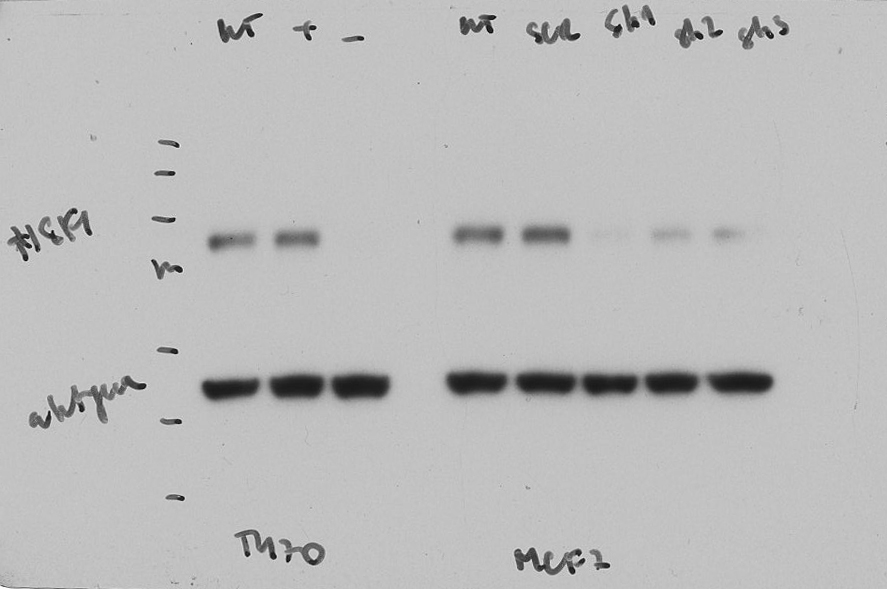

Supplement: Source data 1. — The original files of the full raw unedited blots and gels and figures with the uncropped blots and gels with the relevant bands labeled. [file elife-69843-supp9.zip › Figure 1-figure supplement 1A left actin.jpg]

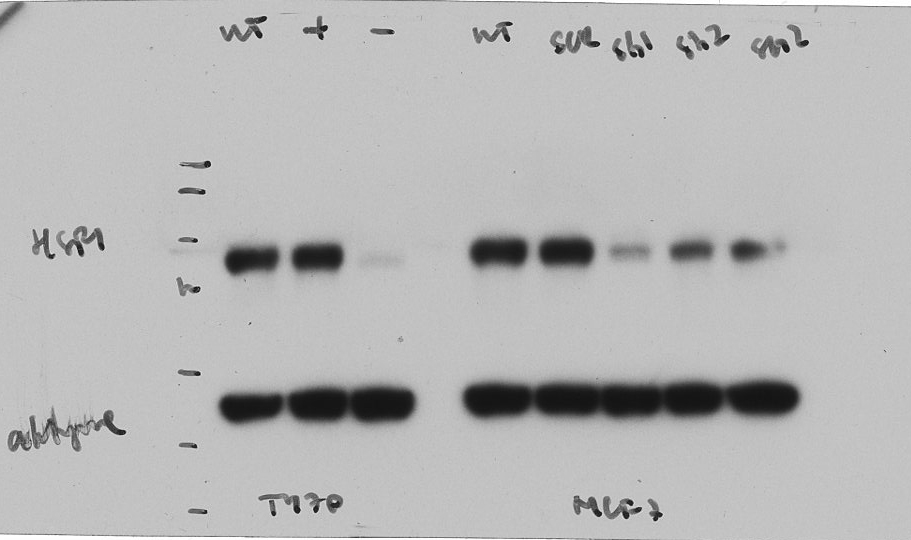

Supplement: Source data 1. — The original files of the full raw unedited blots and gels and figures with the uncropped blots and gels with the relevant bands labeled. [file elife-69843-supp9.zip › Figure 1-figure supplement 1A left HSF1.jpg]

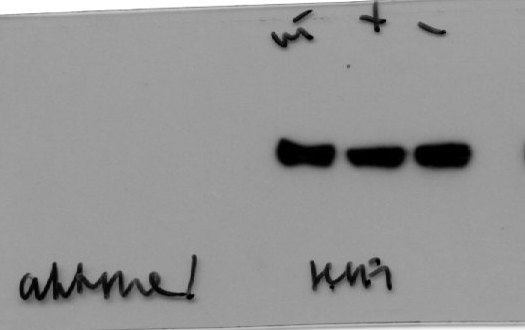

Supplement: Source data 1. — The original files of the full raw unedited blots and gels and figures with the uncropped blots and gels with the relevant bands labeled. [file elife-69843-supp9.zip › Figure 1-figure supplement 2A actin.jpg]

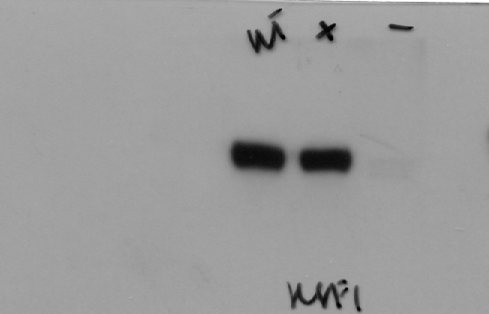

Supplement: Source data 1. — The original files of the full raw unedited blots and gels and figures with the uncropped blots and gels with the relevant bands labeled. [file elife-69843-supp9.zip › Figure 1-figure supplement 2A HSF1.jpg]

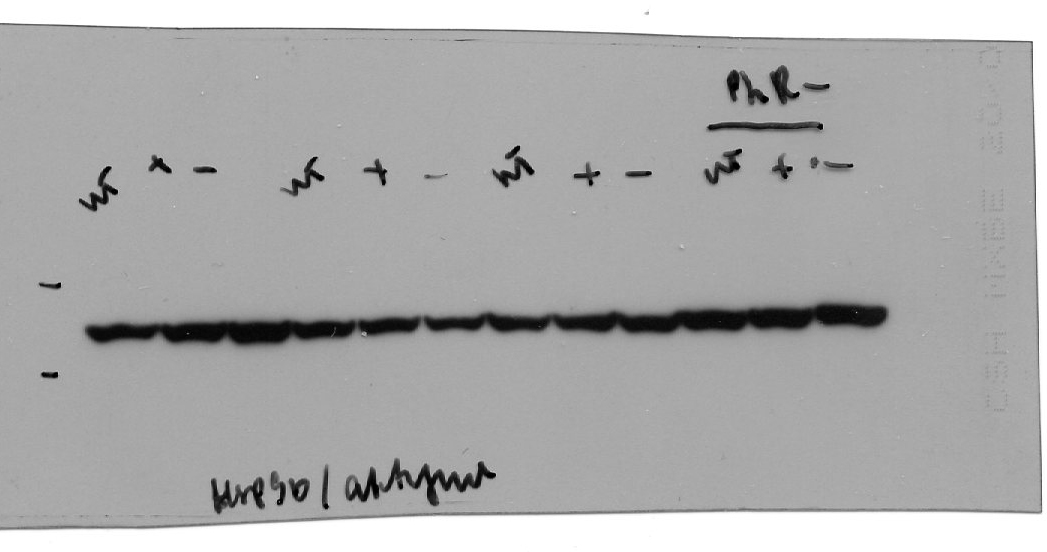

Supplement: Source data 1. — The original files of the full raw unedited blots and gels and figures with the uncropped blots and gels with the relevant bands labeled. [file elife-69843-supp9.zip › Figure 3-figure supplement 2B MCF7 left actin to HSP90.jpg]

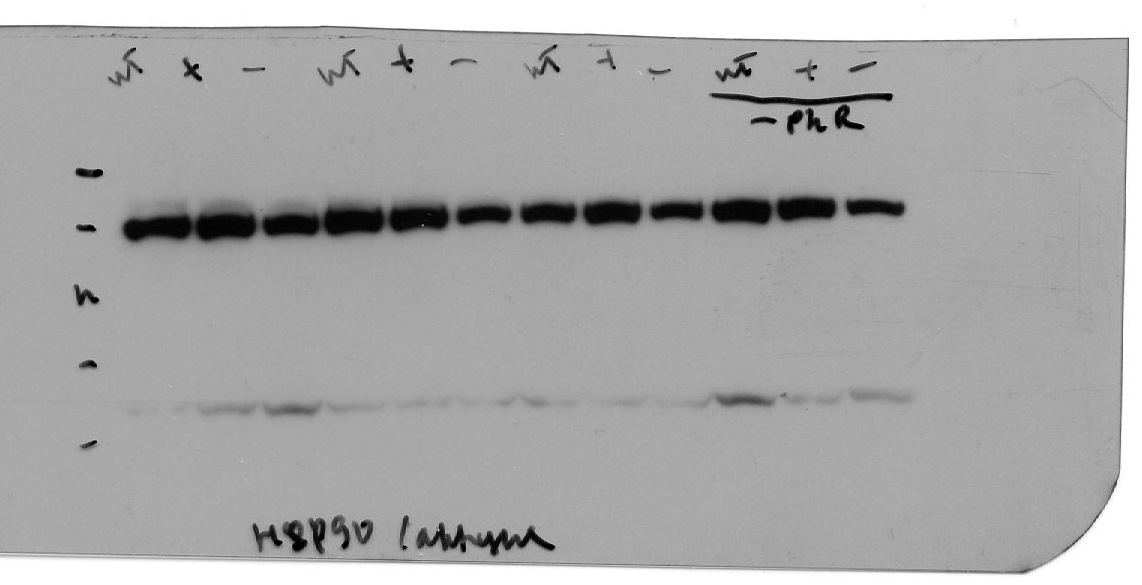

Supplement: Source data 1. — The original files of the full raw unedited blots and gels and figures with the uncropped blots and gels with the relevant bands labeled. [file elife-69843-supp9.zip › Figure 3-figure supplement 2B MCF7 left HSP90.jpg]

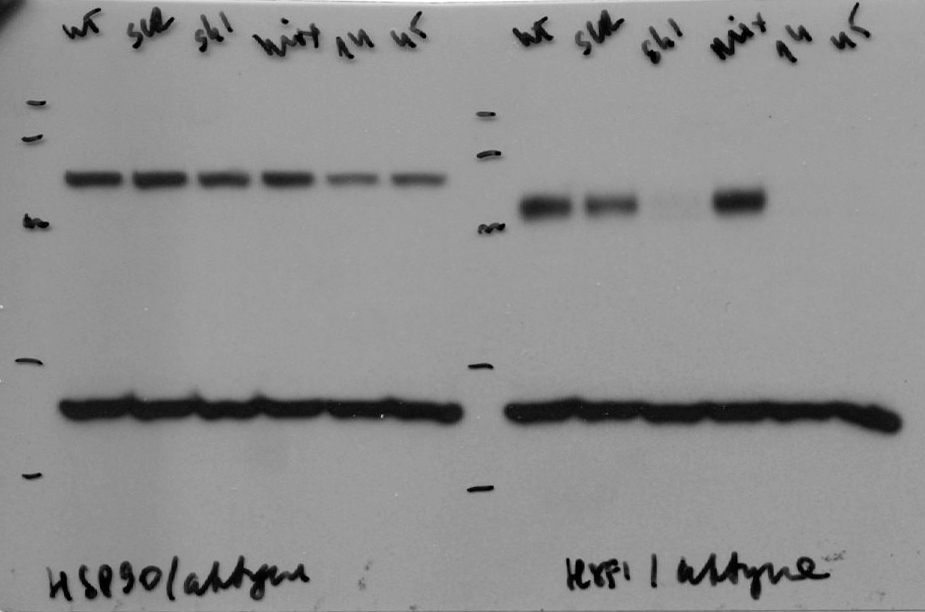

Supplement: Source data 1. — The original files of the full raw unedited blots and gels and figures with the uncropped blots and gels with the relevant bands labeled. [file elife-69843-supp9.zip › Figure 3-figure supplement 2B MCF7 right HSP90 actin.jpg]

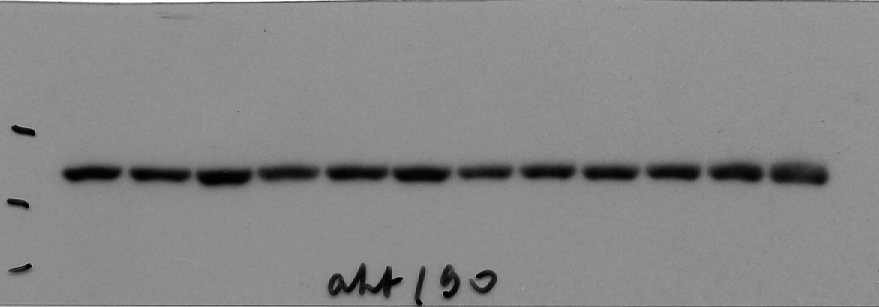

Supplement: Source data 1. — The original files of the full raw unedited blots and gels and figures with the uncropped blots and gels with the relevant bands labeled. [file elife-69843-supp9.zip › Figure 3-figure supplement 2B T47D actin to HSP90.jpg]

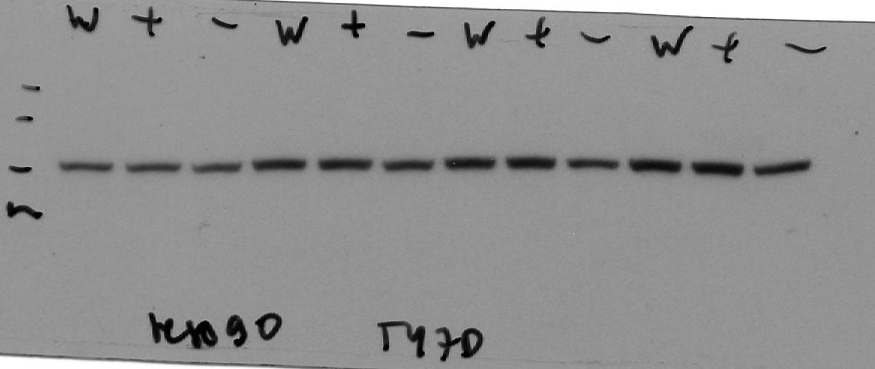

Supplement: Source data 1. — The original files of the full raw unedited blots and gels and figures with the uncropped blots and gels with the relevant bands labeled. [file elife-69843-supp9.zip › Figure 3-figure supplement 2B T47D HSP90.jpg]

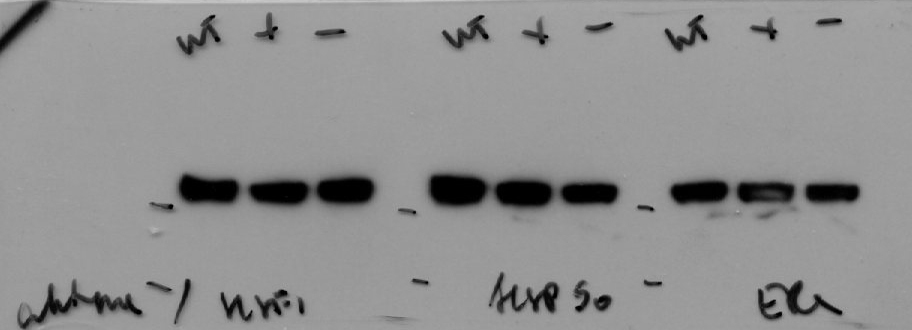

Supplement: Source data 1. — The original files of the full raw unedited blots and gels and figures with the uncropped blots and gels with the relevant bands labeled. [file elife-69843-supp9.zip › Figure 3-figure supplement 2C T47D actin to ERalpha.jpg]

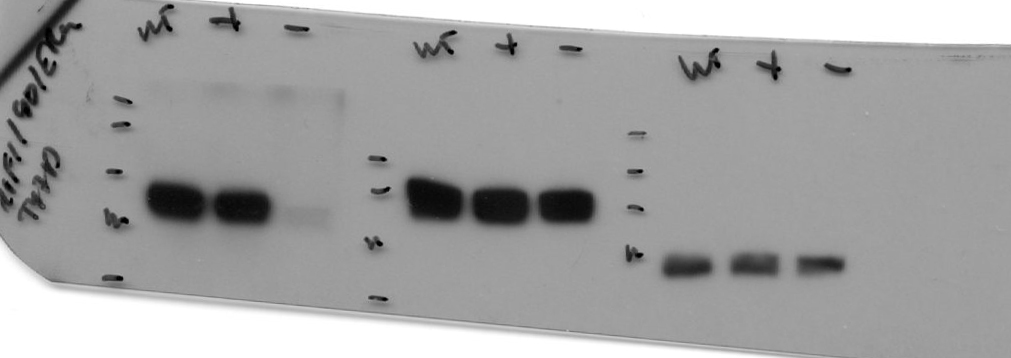

Supplement: Source data 1. — The original files of the full raw unedited blots and gels and figures with the uncropped blots and gels with the relevant bands labeled. [file elife-69843-supp9.zip › Figure 3-figure supplement 2C T47D ERalpha shorter.jpg]
